# Putative Mechanisms for Initial Reaction of Anaerobic Benzene Degradation Presumably Involving a Flavoprotein

**DOI:** 10.1111/1462-2920.70394

**Published:** 2026-07-28

**Authors:** Florin Musat, Lars Wöhlbrand, Rene Jarling, Michael Kube, Niculina Musat, Patrick Becker, Stefan Bruns, Richard Reinhardt, Rudolf Amann, Friedrich Widdel, Heinz Wilkes, Ralf Rabus

**Affiliations:** ^1^ Max Planck Institute for Marine Microbiology Bremen Germany; ^2^ Department of Biology Aarhus University Aarhus Denmark; ^3^ Institute for Chemistry and Biology of the Marine Environment (ICBM) Carl von Ossietzky Universität Oldenburg Oldenburg Germany; ^4^ Institute of Animal Science University of Hohenheim Stuttgart Germany; ^5^ Max Planck‐Genome‐Centre Cologne Cologne Germany

**Keywords:** anaerobic degradation, benzene, cultivation, flavoprotein, genomics, metabolite analyses, proteomics

## Abstract

Biodegradation of benzene is most challenging among the plethora of hydrocarbons due to its high chemical stability. While aerobic bacteria employ O_2_‐dependent oxygenases to overcome the high energy barrier, anaerobic benzene degradation remains enigmatic. Here we achieve an anaerobic enrichment, which is clonal with respect to the benzene‐degrading, sulphate‐reducing, marine bacterium BzS1, from a previously reported enrichment culture. We investigate this clonal enrichment culture by metabolite and proteogenomic analyses. BzS1 has a tailor‐made catabolic network for utilising benzene, toluene, and acetate. Concerning the initial reaction of anaerobic benzene degradation, labelling experiments render previously suggested direct carboxylation (to benzoate) or methylation (to toluene) unlikely. Accordingly, a benzoate‐CoA ligase is not encoded in the genome and proteins of anaerobic toluene degradation are not detectable in benzene‐utilising cells. Rather, detection of labelled 3‐phenylpropanoate hints at addition of benzene to a C_3_‐cosubstrate. Moreover, a presumptively heterotrimeric flavoprotein is present at remarkably high abundance (~⅕ of covered proteome) during anaerobic growth with benzene. This protein is related to glycolate oxidase and flavoproteins of the VOA/PCMH family. Taken together, these findings open the possibility of considering flavin cofactor involvement for the initial reaction using, for example, enolpyruvate (central metabolite) or glutaconyl‐CoA (intermediate of benzoyl‐CoA pathway) as cosubstrate.

## Graphical abstract

Anaerobic biodegradation of benzene by sulphate‐reducing strain BzS1 was studied by combining genomics, differential proteomics and targeted metabolite analyses. Strain BzS1 has an exceptionally streamlined metabolism. A highly abundant heterotrimeric flavoprotein may add benzene to either enolpyruvate or glutaconyl‐CoA followed by channelling into the central benzoyl‐CoA pathway.

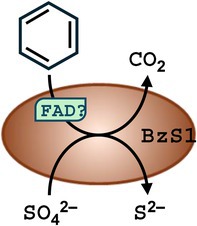

## 1 Introduction

The crucial role of sulphate‐reducing bacteria (SRB) in remineralising organic carbon (C_org_) in marine sediments and thus in linking the global carbon and sulphur cycles is long recognised (Jørgensen 1982; Widdel 1988). SRB are currently accounted for an annual turnover of approx. 77 Tmol C_org_ (Jørgensen et al. 2024), despite their metabolism operating under certain conditions close to the thermodynamic limit (Thauer et al. 1977). This remarkable activity requires the capacity of complete substrate oxidation (to CO_2_), which typifies members of the family *Desulfobacteraceae*. These degrade a large variety of organic compounds ranging from simple fermentation end products and long chain fatty acids to aromatic compounds and hydrocarbons (e.g., Widdel 1988; Rabus et al. 2013). A recent comparative proteogenomic study linked congeneric catabolic‐regulatory networks to the ecophysiological impact of these SRB (Wöhlbrand et al. 2025). The anaerobic degradation of hydrocarbons by SRB related to *Desulfobacteraceae* even from crude oil (Rueter et al. 1994) agrees with the occurrence of SRB in prolific hydrocarbon basins such as the Guyamas Basin in the Gulf of California (Dhillon et al. 2003) or hydrocarbon seeps in the Gulf of Mexico (Zhao et al. 2020), but also to their undesired activity in oil reservoirs (Gieg et al. 2011).

Benzene is the archetype of aromatic hydrocarbons, a prominent constituent of crude oil (Tissot and Welte 1984), a core building block in the petrochemical industry with worldwide production exceeding 70 million metric tons (~0.9 Tmol) (https://www.statista.com/statistics/1108114/global‐benzene‐capacity/), and of environmental and health concern due to its documented association with carcinogenesis and cardiovascular disease (Zhou et al. 2025).

The main challenge in anaerobic degradation of aromatic hydrocarbons is to overcome the high energy barrier when cleaving apolar C—H bonds, which is highest for unsubstituted benzene (~460 kJ/mol) (Widdel and Rabus 2001), and coping with the resonance stabilisation of the aromatic ring. The initial reaction of anaerobic alkylbenzene degradation is known to target the alkyl substituent and proceed either via (i) the thiol radical mediated addition to fumarate by arylsuccinate synthases or (ii) the O_2_‐independent hydroxylation by molybdenum cofactor‐employing, water‐dependent alkyl dehydrogenases (WADHs) (for overview Rabus et al. 2016). By contrast, anaerobic biodegradation of unsubstituted aromatic hydrocarbons is far less well understood. In the case of naphthalene, multi‐layered evidence suggests initial conversion to 2‐naphthoate by a novel carboxylase employing a prenylated flavin mononucleotide cofactor (e.g., Mouttaki et al. 2012; Payne et al. 2015; Heker et al. 2023). Anaerobic degradation of benzene was demonstrated in microcosms and enrichment cultures under various electron acceptor conditions including nitrate, Fe(III), and sulphate reduction as well as methanogenesis (for overview, see Meckenstock et al. 2016). Insights into anaerobic benzene activation were initially obtained from labelling/metabolite studies, leading to three proposed reactions: (i) hydroxylation to phenol (Grbić‐Galić and Vogel 1987; Caldwell and Suflita 2000), (ii) direct carboxylation to benzoate (Caldwell and Suflita 2000), and (iii) methylation to toluene (Coates et al. 2002; Ulrich et al. 2005). It should be noted that genomic (Salinero et al. 2009) and dual isotopic (Fischer et al. 2008) analyses have already questioned hydroxylation (followed by carboxylation) as a mode of anaerobic activation of benzene. While direct carboxylation of benzene was supported by metatranscriptomic analysis of a nitrate‐reducing enrichment culture (Luo et al. 2014), it (as well as methylation) was rather excluded by analysis of the metagenome‐assembled genome (MAG) of *Pelotomaculum* ca. BPL obtained from a sulphate‐reducing enrichment culture originating from a former coal gasification site (Poland) (Dong et al. 2017). Most recently, proteogenomic investigations (Toth et al. 2026) of a methanogenic, benzene‐degrading enrichment culture, originating from hydrocarbon‐contaminated soil/aquifer (USA) (Toth et al. 2021), revealed abundant formation of several proteins (termed ‘Magic’ proteins) in *Desulfobacterota*‐related members (viz. *Ca*. Anaerobenzenivorax ORM2) of the culture. The coding ‘Magic’ gene cluster was also detected in MAGs originating from hydrothermal systems and the aforementioned *Pelotomaculum* ca. BPL. Toth et al. (2026) speculated that these ‘Magic’ proteins are involved in benzene activation, possibly using glutaconyl‐CoA as cosubstrate, and that ultimately acetyl‐CoA and cinnamate are formed.

In the present study, we used a previously reported sulphate‐reducing, benzene‐degrading enrichment culture originating from marine sediment of a Mediterranean lagoon (France) (Musat and Widdel 2008) as a starting point to obtain a clonal enrichment culture (benzene‐degrading phylotype BzS1 accounting for 95% of cells) and to investigate the mechanism of anaerobic benzene degradation. We combined anaerobic cultivation, targeted metabolite analyses, genomics, and (differential) proteomics across a five‐step approach (schemed in Figure S1), which, due to the very slow growth and low yields of the cultures, spanned over almost 2 decades. Overall, we (i) reconstruct a tailored catabolic network of BzS1, (ii) provide evidence against the previous hypotheses of benzene carboxylation and methylation, respectively, (iii) also show the abundant formation of a presumptively heterotrimeric flavoprotein (related to aforementioned ‘Magic’ proteins) that could be involved in the initial activation of benzene, and (iv) discuss the suitability of various potential cosubstrates (in particular (enol)pyruvate versus glutaconyl‐CoA) against the backdrop of observed metabolites (including detection of 3‐phenylpropanoate formed from labelled benzene) and mechanistic considerations.

## 2 Experimental Procedures

### 2.1 Cultivation

The enrichment culture was grown with benzene as sole source of organic carbon and energy in defined, strictly anoxic, bicarbonate‐buffered synthetic marine medium with sulphide as reductant (Widdel and Bak 1992; Musat and Widdel 2008). Routine cultivation was done in butyl rubber stopper‐sealed 200‐mL flat glass bottles with 100 mL medium, 10 mL inoculum, and a non‐toxic benzene solution (0.5% v/v) in 5 mL overlaid, anoxic 2,2,4,4,6,8,8‐heptamethylnonane (HMN). The headspace was N_2_‐CO_2_ (9:1 v/v). Cultures were incubated at 28°C with slow horizontal shaking (50 rpm) while contact of the hydrocarbon phase with the stopper was avoided (Rabus et al. 1993). Strains were isolated via anoxic agar and liquid dilution series (Widdel and Bak 1992) using benzene (0.5% v/v in HMN) or benzoate (5 mM) as substrate. Active sulphate reduction was detected by photometric quantification of sulphide via formation of colloidal copper sulphide (Cord‐Ruwisch 1985). Consumption of provided benzene was determined by gas chromatography‐flame ionisation detector (GC‐FID). Sulphate reduction rates (SRR) were calculated from the linear section of sulphide formation profiles of the original and the clonal enrichment culture, respectively. All used chemicals were of analytical grade.

For proteomic analyses, routine BzS1 cultures were harvested as previously described (Gade et al. 2003; Wöhlbrand et al. 2007). Essentially, the HMN‐phase was removed using a separatory funnel and the obtained cell suspension centrifuged (10,000 × g, 20 min, 4°C). After washing with Tris‐buffer (100 mM Tris–HCl pH 7.5, 5 mM MgCl_2_), cells were snap frozen in liquid nitrogen and stored at −80°C until further analyses.

For metabolite extraction, BzS1 cultures were established in 200‐mL flat glass bottles containing 100 mL medium supplemented with 5 mM sulphate (instead of 28 mM used for routine growth) and 3 mL L^−1^ trace element solution (Musat and Widdel 2008). Bottles were inoculated with 0.5 mL of a 20‐fold concentrated BzS1 cell suspension, which was beforehand generated by centrifugation of active cultures under an anoxic (N_2_) headspace. For labelling experiments, benzene was supplied dissolved in HMN (0.5% v/v), either as unlabelled benzene, (^2^H_6_)benzene (99.5 atom % ^2^H; Sigma‐Aldrich, St. Louis, MO, USA), or (^13^C_6_)benzene (99 atom % ^13^C; Sigma‐Aldrich). In addition, experiments with a binary mixture of unlabelled benzene and fluorobenzene (each at 0.5% v/v in HMN) were conducted. At the end of the growth phase, cultures were prepared for metabolite analyses as described before (Rabus et al. 2001). Essentially, cultures were first inactivated by heating for 20 min in a water bath at 80°C. Then, the HMN phase was removed using separatory funnels, and the aqueous phase of each culture was acidified followed by three rounds of extraction with diethylether (100, 50, and 50 mL). The diethylether extracts were combined, dried over anhydrous sodium sulphate, and stored in 100‐mL glass bottles sealed with screw‐on lids with Teflon gaskets.

### 2.2 Phylogenetic Analysis and Cell Hybridisation

The 16S rRNA gene of the isolated strains was amplified by PCR and sequenced using an ABI Prism BigDye Terminator v 3.0 cycle sequencing kit and an ABI Prism 3100 Genetic Analyser (Applied Biosystems, Waltham, MA, USA), while others were retrieved from public databases. The (near) full‐length 16S rRNA gene sequences (> 1400 bp) were imported into ARB (v6.0.6) (Ludwig et al. 2004) aligned against the SILVA release 138 database (Quast et al. 2013) using the integrated SILVA alignment framework implemented in ARB. Following manual curation, phylogenetically relevant sequences and outgroups were selected within ARB, and the resulting alignment was exported as aligned FASTA files. Maximum‐likelihood phylogenetic trees were reconstructed using IQ‐TREE v2.1.4 (Minh et al. 2020) using ModelFinder for substitution model selection and ultrafast bootstrap approximation (1000 replicates). Final trees were visualised, rooted, annotated, and collapsed for presentation in iTOL v7.6 (Letunic and Bork 2024).

For 16S rRNA‐targeted whole‐cell hybridisation, cells from the enrichment culture and isolates were fixed for 3 h at a final concentration of 2% (w/w) formaldehyde at 4°C, washed twice with 1 × phosphate‐buffered saline (PBS) and stored in 1 × PBS‐ethanol (1:1) at ~20°C. Fixed cells were transferred onto 0.2 μm pore‐sized polycarbonate filters (filter code: GTTP; Millipore, Billerica, MA, USA), hybridised with Cy3‐labelled oligonucleotide probes (Biomers, Ulm, Germany), additionally stained with 4′,6′‐diamidino‐2‐phenylindole (DAPI) and microscopically counted (Snaidr et al. 1997). An oligonucleotide probe (DTIG143, 5′‐TTCGAAGGGTTATCCCGG; 20% formamide) targeting *Desulfotignum* species including strains BtS1 and BtS30 (commensal benzoate utilisers, incapable of degrading benzene), was designed using the ARB software (Ludwig et al. 2004) and evaluated in hybridisation assays with increasing formamide concentrations using the targeted isolated strains as positive controls and *Desulfuromonas acetexigens* (one mismatch) as a negative control. Other oligonucleotide probes used were BZN649 (targeting the BzS1 phylotype; Musat and Widdel 2008), DSS658 (for the *Desulfosarcina* clade; Manz et al. 1998), EUB338 (targeting most Bacteria), and NON338 (nonsense probe; Amann et al. 1990) (Table S1).

### 2.3 Mass Spectrometic Analyses of Individual and Bulk Cells

For secondary ion mass spectrometric analyses of individual cells (nanoSIMS), 0.8 L of an active enrichment culture was centrifuged under a N_2_ headspeace. Cells were re‐suspended in 100 mL anoxic medium. They were first incubated with non‐labelled benzene to verify activity. After sulphide production had ceased, a mixture of unlabelled and (^13^C_6_)benzene (2:1) was added and resuming sulphide production was monitored. Parallel sub‐samples were withdrawn at defined time points for determination of bulk ^13^C‐incorporation and for SIMS analyses. Bulk ^13^C‐incorporation was measured in 0.5 mL sub‐samples on carbon‐free glass‐fibre filters, by flash combustion in excess oxygen at 1050°C in an automated elemental analyser (Thermo Flash EA, 1112 Series; ThermoFisher Scientific Inc., Waltham, MA, USA) coupled to a Delta Plus Advantage mass spectrometer (Thermo Finnigan, Waltham, MA, USA). For nanoSIMS analyses, cells were fixed (see Section 2.2), added on gold–palladium‐coated GTTP filters, and hybridised with horse radish peroxidase‐labelled oligonucleotide probes followed by deposition of fluorine‐containing tyramides (HISH‐SIMS; Musat et al. 2008). The analysis was performed using a NanoSIMS 50 L instrument (Cameca, Gennevilliers, France). For each individual cell, we recorded simultaneously secondary ion images of ^12^C, ^13^C, ^14^N and ^19^F using four electron multipliers. Measurements as well as image and data processing were performed as described earlier (Musat et al. 2008).

### 2.4 Genomics

#### 2.4.1 Genome Library Construction, Sequencing and Analyses

The original enrichment culture (2 L) as well as the here obtained clonal enrichment culture (2 L), both of which dominated by phylotype BzS1, were freed from the organic carrier phase by means of a separatory funnel and centrifuged (10,000 × g, 20 min, 4°C) in preparation for (meta)genomic analyses. The complete genome of BzS1 was determined for each of the two stages of enrichment according to the same procedure yielding identical sequences. Essentially, cells were lysed with sodium ethylenediaminetetraacetate, cetyltrimethylammonium bromide and sodium dodecyl sulphate as described (Rabus et al. 2002). Fragments (1.5–3.5 kbp) generated from genomic DNA by ultrasonication were end‐repaired, size‐selected, ligated in pUC19 vector (Fermentas, St. Leon‐Rot, Germany), propagated in *E. coli* , amplified by PCR and sequenced as described (Rabus et al. 2002, 2005) using an ABI3730 capillary sequencer system (ABI, Weiterstadt, Germany), yielding an 11‐fold coverage. Gaps and regions of weak quality were worked on by primer walking and resequencing, respectively. Sequence data quality was examined by PHRED (Ewing and Green 1998; Ewing et al. 1998). Sequences were assembled with phrap2gap (http://www.sanger.ac.uk/Software7sequencing/docs/phrap2gap/) and GAP4 of the Staden Package (Staden et al. 2000). Sequence data quality is < 1 error per 100,000 bases.

Open reading frames (ORFs) were predicted with ORPHEUS (Frishman et al. 1998). Prediction was refined using ARTEMIS (Rutherford et al. 2000). Translated nucleotide sequences were screened against the SWISSPROT, TREMBL and PIR databases. Similarity searches were carried out using BLAST (Altschul et al. 1997). The possibility that predicted gene products are membrane proteins was assessed by determining the grand average of hydropathicity (GRAVY; Kyte and Doolittle 1982), by predicting the transmembrane segments (TMS) using the TMHMM program (Sonnhammer et al. 1998), and by predicting signal peptides using the SignalP 3.0 program (Bendtsen et al. 2004). Functional prediction was improved by Prokka (Seemann 2014) and comparison with the manually annotated genome of versatile *Desulfosarcina variabilis* 3be13 (Wöhlbrand et al. 2025). This Whole Genome Shotgun project has been deposited at DDBJ/ENA/GenBank under the accession JBVNVM000000000. The version described in this paper is version JBVNVM010000000.

Phylogenetic affiliations of selected proteins (flavoprotein subunits, TetR‐like regulator) were determined and visualised using the integrated Phylogeny.fr platform. The implemented ‘pipeline is already set up to run and connect well‐recognised programs: MUSCLE for multiple alignment, Gblocks for automatic alignment curation, PhyML for tree building and TreeDyn for tree drawing’ (Dereeper et al. 2008).

#### 2.4.2 Comparative Genomics

The translated genomes of BzS1 (this study), *Desulfobacula toluolica* Tol2^T^ (FO203503; Wöhlbrand et al. 2013), *Desulfococcus multivorans* 1be1 (CP015381; Dörries et al. 2016), *Desulfonema limicola* (CP061799; Schnaars et al. 2021), *Desulfonema magnum* (CP061800; Schnaars et al. 2021), *Desulfosarcina aeriophaga* BuS5 (CP087953; Chen et al. 2022), *Ds. variabilis* 3be13 (CP159846; Wöhlbrand et al. 2025), and *Desulfobacterium autotrophicum* HRM2 (CP001087; Strittmatter et al. 2009) were used for comparative genome analyses, essentially as described by Wöhlbrand et al. (2025). For each genome, the EggNOG database (Huerta‐Cepas et al. 2016) was consulted for orthology prediction and functional categorisation. Genome occupancy was calculated as the relative nucleotide proportion of genes per category (KEGG pathway; Kaneshia et al. 2022). PCA plots were generated using custom Matlab (version 2024a; Mathworks, Natick, MA, USA) code and circle plots were created using the implemented CircularGraph function (Kassebaum 2025, https://github.com/paul‐kassebaum‐mathworks/circularGraph). Thereby, for genes assigned to two or more KEGG pathway module categories, the respective share was equally allotted to the corresponding categories.

### 2.5 Metabolite Analyses

Gas chromatographic‐mass spectrometric (GC–MS) analyses of extracts from experiments with culture BzS1 applying unlabelled and isotope‐labelled benzenes were performed on a Trace GC 2000 gas chromatograph coupled to a DSQ mass spectrometer (both Thermo Scientific, Bremen, Germany). Prior to GC–MS analyses, extracts were evaporated to dryness and dissolved in dichloromethane. Then, they as well as the reference compounds were methylated using diazomethane, which was freshly prepared from Diazald (Sigma‐Aldrich). The gas chromatograph was equipped with a PTV injector in splitless mode and a SGE BPX‐5 fused silica capillary column (50 m length, 0.22 mm inner diameter (i.d.), 0.25 μm film thickness). The oven temperature program started at 50°C, which was held for 1 min and then raised to 310°C, which was held for 30 min, with a heating rate of 3°C min^−1^. Helium was used as carrier gas. The ionisation mode for the mass spectrometer was electron impact at 70 eV. The transfer line temperature was 310°C and the ion source temperature was 230°C. Full‐scan mass spectra were recorded between *m*/*z* 50 and 600 at a scan time of 0.2 s. The reference standards of the fluorinated compounds and the associated extracts were derivatised to their corresponding methyl esters using trimethylsulfonium hydroxide (TMSH) prior to GC–MS analysis. Samples were dissolved in dichloromethane and an aliquot of 0.2 M TMSH in methanol was added. The reaction mixture was briefly mixed and allowed to react at room temperature for 30 min. Derivatised samples were analysed using a Trace GC Ultra gas chromatograph coupled to an ISQ QD mass spectrometer (both Thermo Scientific). The gas chromatograph was equipped with a PTV injector in splitless mode and an Agilent J&W DB‐5 fused silica capillary column (30 m length, 0.25 mm i.d., 0.25 μm film thickness). The oven temperature program started at 60°C, which was held for 1.5 min and then raised to 320°C, which was held for 22 min, with a heating rate of 3°C min^−1^. Helium was used as carrier gas. The ionisation mode for the mass spectrometer was electron impact at 70 eV. The transfer line temperature was 305°C and the ion source temperature was 275°C. Full‐scan mass spectra were recorded between *m*/*z* 50 and 650 at a scan time of 0.2 s.

### 2.6 Proteomic Analyses

#### 2.6.1 Original Enrichment Culture

Initial proteomic analyses of the original enrichment culture followed a two‐track approach as previously described (Gade et al. 2003; Wöhlbrand et al. 2007). (i) Soluble (membrane‐free) cell extracts were prepared as follows: cell breakage was achieved by grinding (sample grinding kit; GE Healthcare, Munich, Germany) in 1 mL lysis buffer (7 M urea, 2 M thiourea, 2% DTT, 2% CHAPS, 0.5% carrier ampholytes (GE Healthcare)) and cell debris was removed by ultracentrifugation (100,000 × g, 1 h, 4°C). Protein content was determined according to Bradford (1976). The soluble fraction was analysed by 2D‐gel electrophoresis, applying 300 μg protein to isoelectric focussing with pH 3–11 gradient gels followed by SDS‐PAGE using the IPGphor and EttanDalt systems, respectively (GE Healthcare). Protein spots were manually excised from at least three replicate gels stained with Coomassie Blue, subjected to tryptic digestion and analysed with a MALDI mass spectrometer (4800 Proteomics Analyser; Applied Biosystems). Database queries for peptide fingerprint masses were performed using the Mascot Server (Matrix Science Ltd., London, UK). (ii) The membrane protein‐enriched fraction was prepared as previously described (Nouwens et al. 2000; Trautwein et al. 2012; Koßmehl et al. 2013), involving ultracentrifugation of broken cells (100,000 × g, 1 h, 4°C), treatment with ice‐cold carbonate, solubilisation with hot (95°C) SDS (10 g L^−1^), and determination of protein concentration with the detergent compatible RC DC protein assay (Biorad, Munich, Germany). Separation of proteins (10–50 μg per lane) was achieved by SDS‐PAGE and excised protein bands were digested with trypsin. Then, the peptide mixtures were decomplexed by nanoLC (Ettan MDLC; GE Healthcare) and analysed by a coupled linear ion trap mass spectrometer (Thermo LTQ; Thermo Electron, Waltham, MA, USA). Protein identification was achieved as aforementioned and MS analyses were conducted in 2007–2009 as commissioned work (Toplab GmbH, Planegg, Germany).

#### 2.6.2 Clonal Enrichment Culture BzS1

The clonal enrichment culture BzS1, adapted to anaerobic growth with benzene or toluene, was also subjected to a two‐track proteomic approach: (i) The soluble protein fraction was prepared using urea lysis buffer (30 mM Tris–HCl pH 8.5, 2 M thiourea, 7 M urea) and sample grinding (see Section 2.6.1). Following ultracentrifugation (104,000 × g, 17°C, 1 h), the protein content in the supernatant was determined according to the method described by Bradford (1976) and in‐solution digest performed as reported (Zech et al. 2013). (ii) The membrane protein‐enriched fraction was prepared following cell breakage by means of a french pressure cell at 500 psi essentially as described above (Section 2.6.1). Further, the supernatant of the initial ultracentrifugation, separating the membrane pellet, was additionally analysed for soluble proteins.

Similar amounts of total protein (i.e., 20 μg) per subcellular fraction and substrate condition were separated using large (20 cm) SDS‐PAGE gels. Each lane was cut into 8 gel slices, each of which was further chopped into small pieces and subjected to in‐gel digest as described (Zech et al. 2013). Generated peptide mixtures were decomplexed by nanoLC (UltiMate3000 nanoLC system; ThermoFisher Scientific) applying a trap‐column setup (PepMap nanoTrap: C_18_ 100 Å, 3 μm bead size, 75 μm i.d., 2 cm length; ThermoFisher Scientific) with 0.05% TFA as solvent prior to separation with an analytical column (PepMapRSLC: C_18_ 100 Å, 2 μm bead size, 75 μm i.d., 15 cm length; ThermoFisher Scientific) with a 90 min and a 240 min gradient for membrane protein‐enriched and soluble fractions, respectively. The effluent was continuously analysed by an online coupled ion trap mass spectrometer (amaZon ETD; Bruker Daltonics GmbH, Bremen, Germany). A distal coated fused SilicaTip (NewObjective, Woburn, MA, USA) with an inner diameter of 10 μm was used for ionisation at a voltage of 1.6 kV. Replicate measurements were performed with an advanced ion trap mass spectrometer (amaZon speed ETD; Bruker Daltonics GmbH) using the captive spray ion source (Bruker Daltonik GmbH) with 1.3 kV. For both instruments, the MS method consisted of a full MS scan (mass range 300–1500 *m*/*z*) with subsequent MS/MS of the 10 or 20, respectively, most intense MS peaks (successive exclusion after 1 spectrum for 0.2 min). Protein identification was performed using an in‐house Mascot server searching against a genomic database of BzS1, translated into amino acid sequences applying a target‐decoy strategy and the following settings: Taxonomy: all entries, Enzyme: trypsin, May missed cleavage: 1, Fixed modification: carbamidomethyl (C), Variable modification: oxidation (M), Peptide mass tolerance: 0.4 Da, Fragment mass tolerance: 0.4 Da, Mass values: monoisotopic, Instrument type: ESI‐TRAP and inclusion of peptide Decoy. A false discovery rate (FDR) of < 1% was applied as threshold of significance.

The mass spectrometry proteomics data have been deposited to the ProteomeXchange Consortium via the PRIDE (Perez‐Riverol et al. 2025) partner repository with the dataset identifier PXD080844, and at the FAIRDOMHub repository (Wolstencroft et al. 2017) under the link https://doi.org/10.15490/fairdomhub.1.investigation.823.1.

Comparative analyses of the proteomic datasets were performed using combined peptide count data of all soluble and membrane fractions per substrate condition. A non‐redundant list of detected proteins was created by including only proteins detected in at least two replicate samples. For both, the soluble and the membrane protein‐enriched fractions, average peptide counts were calculated per condition and only the highest peptide count (i.e., either soluble or membrane protein‐enriched fraction) was considered in subsequent analyses, yielding identification of in total 586 unique proteins. Peptide count data as well as unfiltered, detailed protein identification data for all replicate samples (in total 24) is provided in Table S2 (and the tabs therein). Peptide counts of protein constituents of the tailored catabolic network are provided in Table S3.

## 3 Results

### 3.1 Benzene‐Degrading Clonal Enrichment Culture BzS1

The dominant phylotype (originally termed BznS295, here renamed BzS1) in the benzene‐degrading, original enrichment culture affiliated with a cluster comprising other anaerobic degraders of aromatic hydrocarbons within the Deltaproteobacteria: e.g., *m*‐xylene‐degrading strain mXyS1 (Harms et al. 1999), naphthalene‐degrading strain NaphS2 (Galushko et al. 1999), and clones from a benzene‐degrading, sulphate‐reducing consortium (Phelps et al. 1998). The recently reported *Ca*. Anaerobenzenivorax ORM2 (Toth et al. 2026) affiliates with a clearly separated cluster including, e.g., clones from crude‐oil polluted sediment (Figure S2A). Renewed hybridisations with specific oligonucleotide probes (Table S1) showed that BzS1 accounted for 87% of all cells (Figure S2B), consistent with previous observations (Musat and Widdel 2008).

Incubation of the original enrichment culture with (^13^C_6_)benzene followed by HISH‐SIMS demonstrated strong ^13^C incorporation by BzS1 cells after 24 h, while accompanying bacteria showed only minor incorporation (Figure S2C). This confirmed the direct involvement of BzS1 in anaerobic degradation of benzene. Attempts to further purify BzS1 using benzoate yielded benzoate‐degrading strains that were phylogenetically distinct from BzS1, collectively accounted for only 6% of the enrichment culture cells (Figure S2AB) and were unable to anaerobically grow with benzene (Figure S3).

Since no other substrates supported growth of the enrichment culture (Supporting Information, Page 1), isolation was attempted directly with benzene. The resulting colonies consisted of benzene utilisers phylogenetically matching phylotype BzS1, as determined by PCR amplification and sequencing of the 16S rRNA gene. One isolate was selected for subcultivation and further investigations (phylogenetic affiliation as shown in Figure S2A). The culture contained a low‐abundance spirochaete‐like microorganism (~5% of total cells), with a small biovolume (~⅕ of BzS1 cells, contributing merely 1% to the total culture biovolume). Growth of the spirochaete was never promoted in substrate utilisation tests with other compounds, suggesting reliance on minor organic exudates rather than involvement in anaerobic benzene degradation. Taken together, benzene‐utilising BzS1 represents a clonal enrichment culture that is axenic with respect to sulphate reduction (Figure S4A) and excreted minor amounts of benzoate during anaerobic growth with benzene (Figure S4B). For clarity, we use the term ‘culture BzS1’ in the context of cultivation experiments versus ‘BzS1’ when referring to proteogenomic data.

Growth performance of culture BzS1 with benzene was markedly improved compared to the original enrichment culture as demonstrated by calculated average sulphate reduction rates: 0.44 ± 0.09 mmol L^−1^ day^−1^ (*n* = 2 biological replicates) versus 0.19 ± 0.03 mmol L^−1^ day^−1^ (*n* = 8 biological replicates). As expected, culture BzS1 did not grow with benzoate. However, anaerobic growth with toluene began after a lag phase of 1 month, as already observed with the original enrichment culture. Culture BzS1 utilised acetate also, proving its capability for terminal oxidation and complete benzene mineralisation. Other tested substrates were not utilised (Supporting Information, Page 1).

### 3.2 Metabolite Analyses of Cultures Provided With Labelled Compounds

**FIGURE 1:**
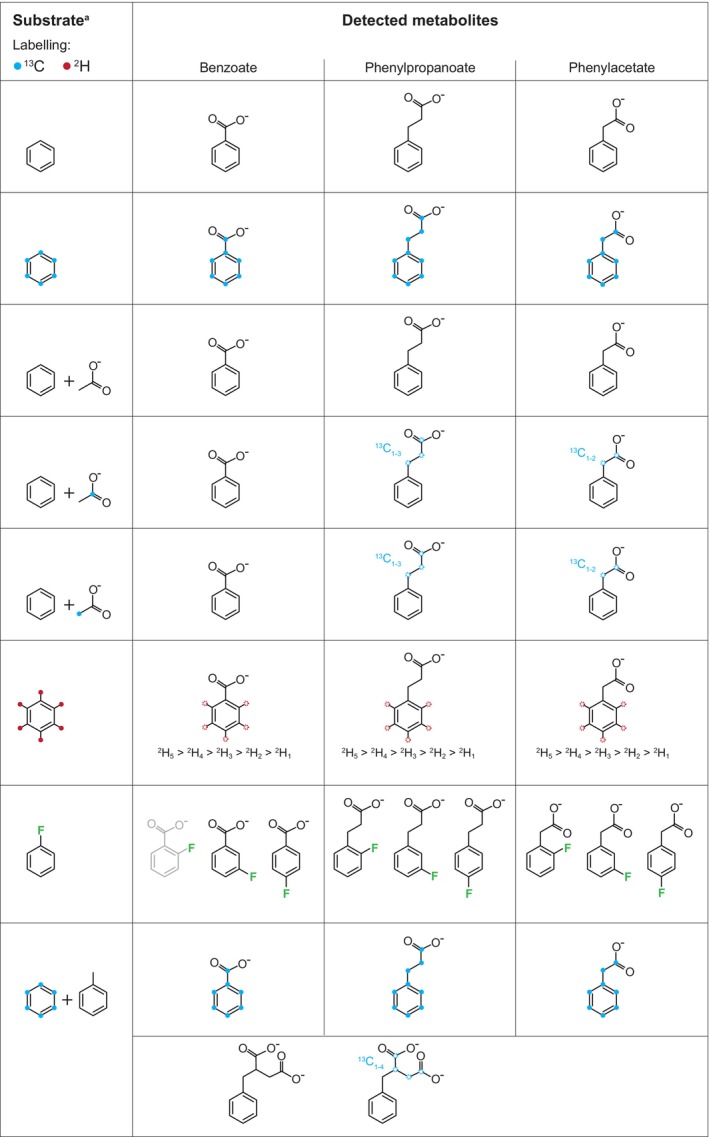
Detected metabolites in benzene‐utilising cultures of sulphate‐reducing culture BzS1. Underlying mass spectrometric or chromatographic data are compiled in the Figures S5–S10.

**FIGURE 2:**
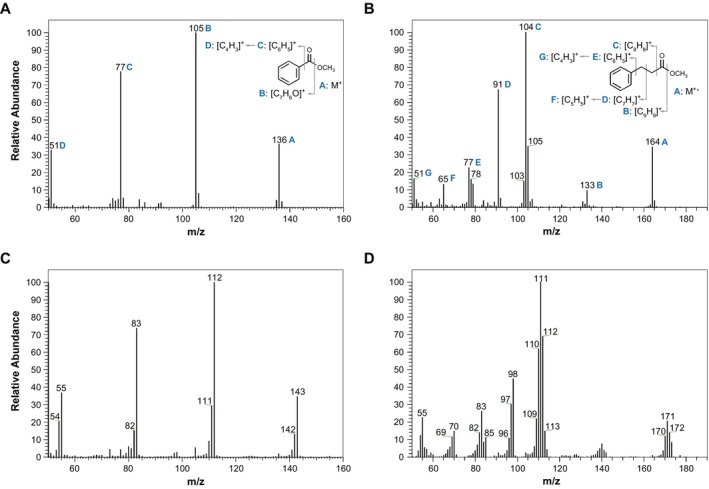
Mass spectra showing the labelling patterns of (^13^C_7_)benzoate (C) and (^13^C_9_)phenylpropanoate (D) (as methyl esters after derivatisation with diazomethane) detected in extracts of BzS1 cultures upon anaerobic growth with (^13^C_6_)benzene. For comparison, mass spectra of the unlabelled reference compounds, including the respective fragmentation patterns, are shown in (A, B).

To obtain metabolite‐inspired insights into the initial reaction(s) of anaerobic benzene degradation, we incubated culture BzS1 with isotope labelled benzene as well as with binary mixtures of benzene and isotope labelled compounds, toluene or fluorobenzene, using cultures growing solely with unlabelled benzene as reference. An overview of these experiments is illustrated in Figure 1 and underlying mass spectrometric details are exemplified in Figure 2 and comprehensively provided together with chromatographic data in Figures S5–S10.

#### 3.2.1 ( ^13^C_6_ )Benzene

Utilisation of (^13^C_6_)benzene by culture BzS1 resulted in the formation of ^13^C‐labelled benzoate, phenylacetate and 3‐phenylpropanoate, with labelling patterns recognisable by the molecular ion cluster and clusters of specific fragment ions (Figure 2 and Figures S5–S7). In benzoate, M^+^ was shifted from *m*/*z* 136 to 142 and 143, and the fragment ion [C_7_H_5_O]^+^ was shifted from *m*/*z* 105 to 111 and 112, revealing 6 or 7 ^13^C atoms in benzoate. The ratio of signal intensities in the labelled compound indicates that the carboxyl group was ~70% ^13^C‐labelled and ~30% unlabelled. In 3‐phenylpropanoate, the molecular ion cluster appeared at *m*/*z* 170, 171, 172 and 173, corresponding to shifts of 6, 7, 8 and 9 amu, respectively. This indicates that the C_3_ side chain contained 0–3 ^13^C atoms, whereby the isotopologs with one or three ^13^C atom(s) in the side chain (*m*/*z* 171 or 173) produce the strongest and weakest signals, respectively. In the case of phenylacetate, both labelled and unlabelled isotopologs were detected, which documents that part of it is not directly originating from metabolised (^13^C_6_)benzene. In the ^13^C‐labelled portion of phenylacetate, the molecular ion cluster appeared at *m*/*z* 156, 157 and 158, indicating that the C_2_ side chain, similar to the C_3_ side chain in 3‐phenylpropanoate, contained 0–2 ^13^C atoms. Taken together, these results show that the side chains in benzoate, phenylacetate, and 3‐phenylpropanoate did not originate entirely from the supplied (^13^C_6_)benzene. One may speculate that ^12^CO_2_‐fixation via the reversed Wood‐Ljundahl (WL) pathway and/or pyruvate:ferredoxin oxidoreductase (PFOR) could contribute the unlabelled carbon atoms. Such a metabolic scenario was recently described as the chemoorganoautotrophic lifestyle of naphthalene‐utilising, sulphate‐reducing enrichment culture N47 (Heker et al. 2025).

#### 3.2.2 Benzene Plus Acetate, 1‐( ^13^C)Acetate or 2‐( ^13^C)Acetate

When culture BzS1 was provided with either unlabelled acetate, 1‐(^13^C)acetate, or 2‐(^13^C)acetate, benzoate, phenylacetate, and 3‐phenylpropanoate were detected in all cases. When 1/2‐(^13^C)acetate was provided, no label was detected in the carboxyl group of benzoate, which indicates that acetate does not represent a major source for the carboxyl group in formed benzoate. Similar findings were previously reported by Phelps et al. (2001). This apparent absence of label was not due to an inability of acetate uptake, since the detection of a single ^13^C atom in the side chains of phenylacetate and 3‐phenylpropanoate (not assignable to a specific carbon atom) demonstrates that culture BzS1 can import and metabolise exogenous acetate.

#### 3.2.3 ( ^2^H_6_ )Benzene

In the presence of (^2^H_6_)benzene, BzS1 cultures formed (^2^H_5_)benzoate (and also in decreasing amounts (^2^H_4_), (^2^H_3_), (^2^H_2_), (^2^H_1_) and (^2^H_0_)benzoate); formation of (^2^H_0‐4_)benzoate indicates that a partial exchange with external hydrogen had occurred. The mass spectrum presented by Phelps et al. (2001) could be interpreted in a similar way. The hydrogen exchange could be either associated with the mechanism of benzene activation or indicate reversibility of the reaction. For the naphthalene‐degrading enrichment culture N47, the incorporation of 0, 1, 2, or 3 deuterium atoms in naphthoyl‐CoA formed in assays carried out in deuterated buffer has been interpreted such that the carboxylation reaction is fully reversible (Heker et al. 2023).

#### 3.2.4 Fluorobenzene

Providing culture BzS1 with fluorobenzene led to the formation of all three isomeric fluorobenzoates. While similar quantities of 3‐ and 4‐fluorobenzoate were detected, only traces of 2‐fluorobenzoate were found (Figure S8). Considering the *ortho*‐/*para*‐directing effect of the fluorine atom, an electrophilic aromatic substitution as mechanism of benzene activation appears at least questionable. This could open up the theoretical possibility of an aryne mechanism (Wenk et al. 2003), which would be in accord with the observed hydrogen exchange (see Section 3.2.3). However, all three isomeric fluorophenylacetates and fluorophenylpropanoates (Figures S9 and S10) were detected in fairly similar amounts.

#### 3.2.5 ( ^13^C_6_ )Benzene Plus Toluene

Culture BzS1 anaerobically growing with a mixture of (^13^C_6_)benzene and unlabelled toluene formed ^13^C‐labelled benzoate, phenylacetate and 3‐phenylpropanoate as described above (Section 3.2.1), and in addition, unlabelled as well as (^13^C_1–4_)benzylsuccinates, but no unlabelled benzoate. In the case of the benzylsuccinates, the label was exclusively detected in the succinate moiety (mostly in C2 and C3) and not in the benzylic methylene group. This suggests that the fumarate cosubstrate required for benzylsuccinate formation from toluene is partially derived from (^13^C_6_)benzene.

### 3.3 Genome‐Inferred Nutritional Specialisation

**FIGURE 3:**
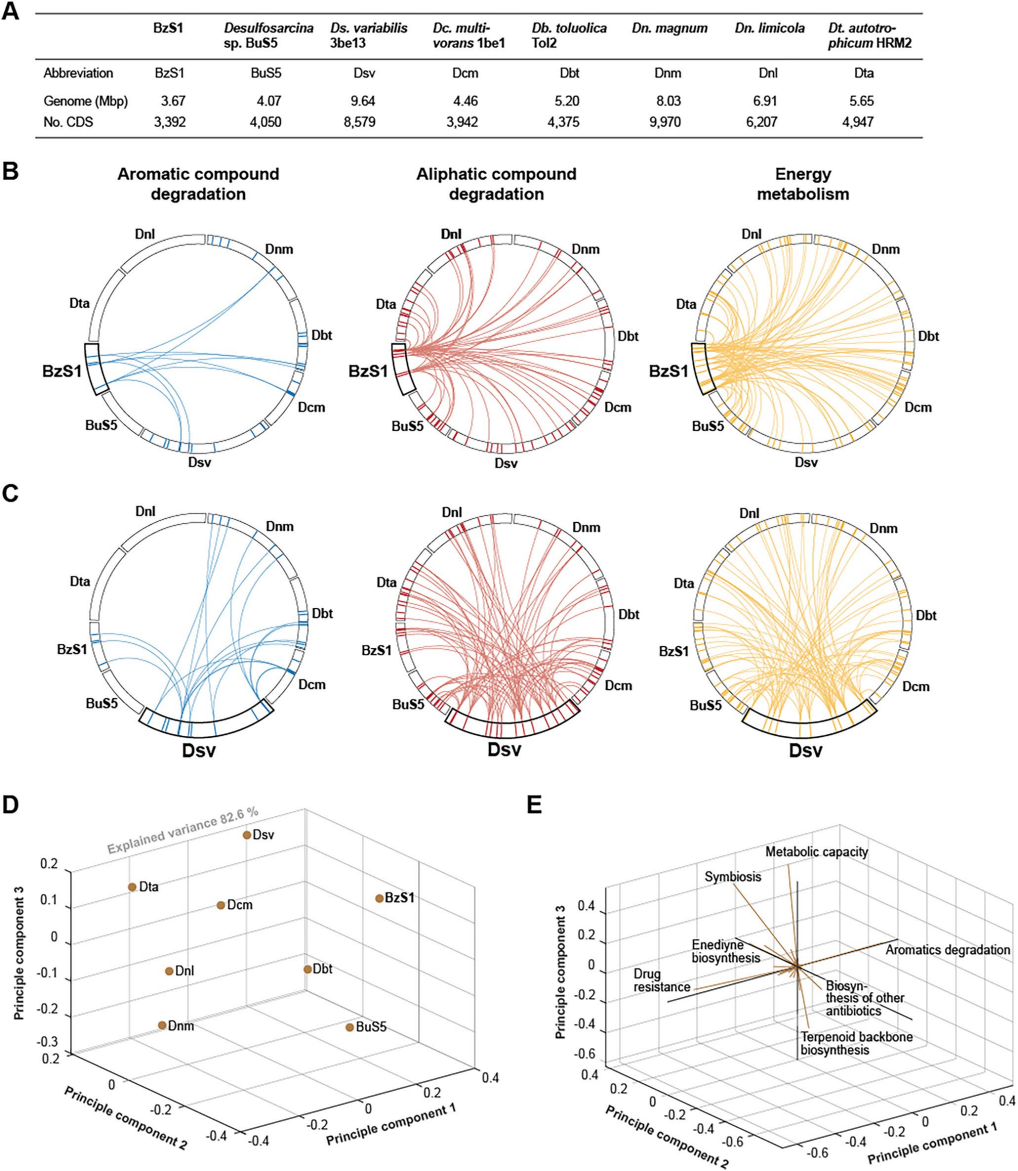
Genomic comparison of BzS1 with other, completely oxidising *Desulfobacteraceae*. (A) Genome size and number of coding sequences of the studied SRB and abbreviations used in subsequent plots. (B, C) Circular graph of scale models of the genomes of the eight studied SRB strains. Positions of ‘metabolic’ gene clusters related to aromatic compound (blue), or aliphatic compound (red) degradation as well as energy metabolism (orange) are indicated. (B) ‘Metabolic’ gene clusters present in specialised BzS1 are connected to corresponding gene clusters in the other studied strains. (C) ‘Metabolic’ gene clusters present in versatile *Ds. variabilis* 3be13 are connected to corresponding gene clusters in the other studied strains. (D) 3D Principal component analysis (PCA) of genomic share of CDSs allocated to KEGG pathway modules. Explained variance is indicated (top). (E) Corresponding PCA loadings plot.

Considering the very limited range of growth substrates of culture BzS1, we explored its comparatively small genome (3.67 Mbp) for the potential to degrade other aromatic compounds. For this purpose, we searched for homologues of respective genes from the model bacteria *Desulfobacula toluolica* Tol2^T^ (sulphate‐reducing; Wöhlbrand et al. 2013) and *Aromatoleum aromaticum* EbN1^T^ (denitrifying; Becker et al. 2024). No (conclusive) genetic makeup for the degradation of the following compounds was found: phenol, phenylacetate, phenylpropanoids, *p*‐cresol, ethylbenzene, acetophenone, and acetone. The genetic equipment for dissimilatory sulphate reduction including associated transmembrane electron transfer complexes corresponds to the generally known composition in SRB (e.g., Barbosa et al. 2024; Wöhlbrand et al. 2025) (Figure S11DE). Taken together BzS1 appears as a true nutritional specialist, which ecophysiologically separates it from the versatile members of the well‐studied and globally occurring family *Desulfobacteraceae* (Wöhlbrand et al. 2025). This observation was augmented by comparative genomics, which revealed (i) the markedly smaller genome size of BzS1 (~2.5‐fold compared to *Ds. variabilis* 3be13; Figure 3A), (ii) its restricted substrate range but similar respiratory equipment compared in particular to highly versatile *Ds*. *variabilis* (Figure 3B,C), and (iii) separation of BzS1 together with *n*‐butane‐degrading, metabolically‐specialised *Ds. aeriophaga* BuS5 (Chen et al. 2022) on a genome‐wide level from the other selected *Desulfobacteraceae* members (Figure 3D).

### 3.4 Tailored Catabolic Network

**FIGURE 4:**
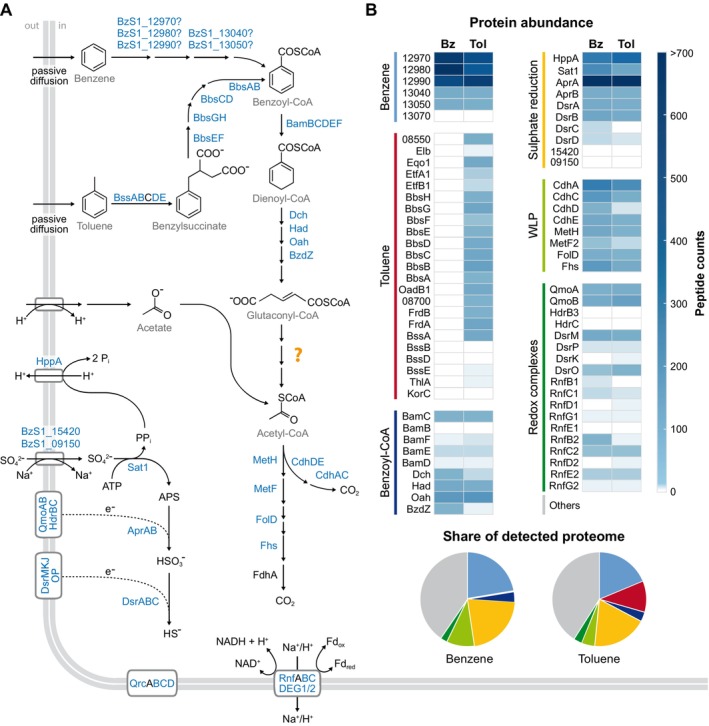
Tailored catabolic network of BzS1 as reconstructed by proteogenomics. (A) Degradation network and dissimilatory sulphate reduction. Details on proteins putatively (indicated by ‘?’) involved in initial benzene activation are illustrated in Figures 5 and 7. Enzyme names (alphabetic order and based on homology to enzymes biochemically characterised in other bacteria): AprAB, adenylylsulphate reductase; BamB–F, anaerobic class II benzoyl‐CoA reductase; BbsAB, benzoylsuccinyl‐CoA thiolase; BbsCD, 2‐[hydroxy(phenyl)methyl]succinyl‐CoA dehydrogenase; BbsEF, benzylsuccinate CoA‐transferase; BbsGH, benzylsuccinyl‐CoA dehydrogenase; BssA–E, benzylsuccinate synthase; BzdZ, predicted dehydrogenase; BzS1_12970/80/90, predicted heterotrimeric flavoprotein putatively involved in hypothesised initial conversion of benzene to 2‐oxo‐3‐phenylpropanoate or 3‐phenylglutaryl‐CoA (see Figure 7); BzS1_13040, predicted 3‐hydroxybutyryl‐CoA dehydrogenase; BzS1_13050, predicted enoyl‐CoA hydratase that together with BzS1_13040 is assumed to function in subsequent conversion to benzoyl‐CoA; BzS1_15420/09150, sodium‐gradient driven sulphate transporter; CdhACDE, CO dehydrogenase; Dch, cyclohexa‐1,5‐diene‐1‐carbonyl‐CoA hydratase; DsrABC, dissimilatory sulphite reductase; DsrMKJOP, dissimilatory sulphate reduction electron transfer complex; FdhA, formaldehyde dehydrogenase; Fhs, formate‐tetrahydrofolate ligase; FolD, bifunctional methylenetetrahydrofolate dehydrogenase/methenyltetrahydrofolate cyclohydrolase; Had, 6‐hydroxycyclohex‐1‐ene‐1‐carboxyl‐CoA dehydrogenase; HdrBC, heterodisulphide reductase; HppA, pyrophosphate‐energised proton pump; MetF, 5,10‐methylenetetrahydrofolate reductase; MetH, 5‐methyltetrahydrofolate methyltransferase; Oah, 6‐oxo‐cyclohex‐1‐ene‐carbonyl‐CoA hydrolase; QmoAB, quinone interacting oxidoreductase; QrcA–D, menaquinone reductase; RnfA–EG, Rnf electron transport complex; Sat1, sulphate adenylyltransferase. (B) Differential proteomic data of benzene‐ versus toluene‐adapted cells. Protein subunits identified by proteomics are marked in blue (details are provided in Table S3). Five‐digit numbers at the heatmap refer to respective locus tags (BzS1_xxxxx) used in subfigure (A).

The limited degradation network is tailor‐made for the three sole growth substrates of BzS1 and schemed in Figure 4A. This network is based on integrated genomic and proteomic analyses. While hydrophobic benzene and toluene should enter the cell simply via passive diffusion, charged acetate requires import by, for example, a H^+^‐driven transporter, which, however, remains unaccounted for at present. Possible reactions for conversion of benzene to the central intermediate benzoyl‐CoA are detailed in the subsequent sections. Toluene degradation should follow the canonical reaction sequence initiated by benzylsuccinate synthase (BssABC), which catalyses the radical addition of toluene to fumarate (Heider et al. 2016; Funk et al. 2014), followed by a modified β‐oxidation pathway leading to benzoyl‐CoA (Bbs proteins) (Weidenweber et al. 2022). The further degradation of benzoyl‐CoA involves ATP‐independent class II benzoyl‐CoA reductase (BamB–F) for initial reductive dearomatisation to a dienoyl‐CoA (Boll et al. 2016), followed by several rounds of modified β‐oxidation via the non‐cyclic 3‐hydroxypimeloyl‐CoA (Breese et al. 1998) to three acetyl‐CoA and CO_2_. Noteworthy, the absence of genes for a known benzoate uptake system (e.g., BenK; Collier et al. 1997) and benzoate‐CoA ligase (BclA; Schühle et al. 2003) explains the incapacity of culture BzS1 to utilise this prominent aromatic compound. Terminal oxidation of acetyl‐CoA to CO_2_ proceeds via the Wood‐Ljungdahl (WL) pathway as typical for members of the completely oxidising family *Desulfobacteraceae* (Wöhlbrand et al. 2025).

The genetic repertoire of BzS1 for the protein complements of dissimilatory sulphate reduction and for associated membrane‐localised electron transfer complexes (Figure 4A, lower part) corresponds to the standard equipment known across many sulphate‐reducing bacteria (Rabus et al. 2015; Wöhlbrand et al. 2025). The only exception is the absent C‐subunit of the membrane‐localised Qmo complex (delivering electrons to APS reductase; Duarte et al. 2016), which appears to be substituted by a heterodisulphide reductase‐like protein (HdrB3C). Such a substitution is known from sulphate‐reducing bacteria (Pereira et al. 2011) and lithotrophic sulphur oxidisers (Appel et al. 2021). In total, 54 of the 62 proteins (87% coverage) assigned to the catabolic network of BzS1 were identified (Table S2).

Taken together, the catabolism of BzS1 is limited as well as specialised, as was previously also reported based on proteogenomic analyses for sulphate‐reducing *Ds*. *aeriophaga* BuS5 specialised in anaerobic degradation of gaseous *n*‐butane (Chen et al. 2022) and most recently found with *Ca*. Anaerobenzenivorax ORM2 (Toth et al. 2026). Apparently, such tailored catabolism is a recurring theme among anaerobic degradation specialists thriving in hydrocarbon‐rich niches.

### 3.5 A Highly Abundant, Conspicuous Flavoprotein

#### 3.5.1 (Differential) Proteomic Detection

A striking, repeated observation from our proteomic analyses was the unusually high abundance of three proteins (BzS1_12970/80/90; predicted sizes of 54.5, 46.5, and 65.8 kDa), which together account for ~⅕ of the detected proteome in benzene‐adapted BzS1 cultures. This share surpasses even the cumulative relative abundance of all identified proteins (17) assigned to dissimilatory sulphate reduction and linked transmembrane electron transfer complexes (22.4% versus 21.8%) (Figure 4B and Table S3). Notably, we recognised the dominance of these proteins already during our initial proteomic analyses of the original benzene‐degrading enrichment culture (Figures S12–S14). Apparently, enzymes for anaerobic hydrocarbon activation are required in high amounts, making up a large proportion of the cellular proteome, as previously also observed, e.g., for *n*‐hexane activating (methylalkyl)succinate synthase in denitrifying *Aromatoleum* sp. strain HxN1 (Grundmann et al. 2008). The BzS1_12970/80/90 proteins were also detected in toluene‐adapted BzS1 cultures, with their cumulative share from the in total resolved proteome being only slightly smaller (18.7% compared to 22.4% in benzene‐adapted cultures). The identified BzS1_12970/80/90 proteins are initially annotated as FAD‐binding oxidase, heterodisulphide subunit C (HdrC)‐like FeS protein, and FAD‐binding oxidase, respectively.

#### 3.5.2 Gene Structures, Organisation and Similarities

**FIGURE 5:**
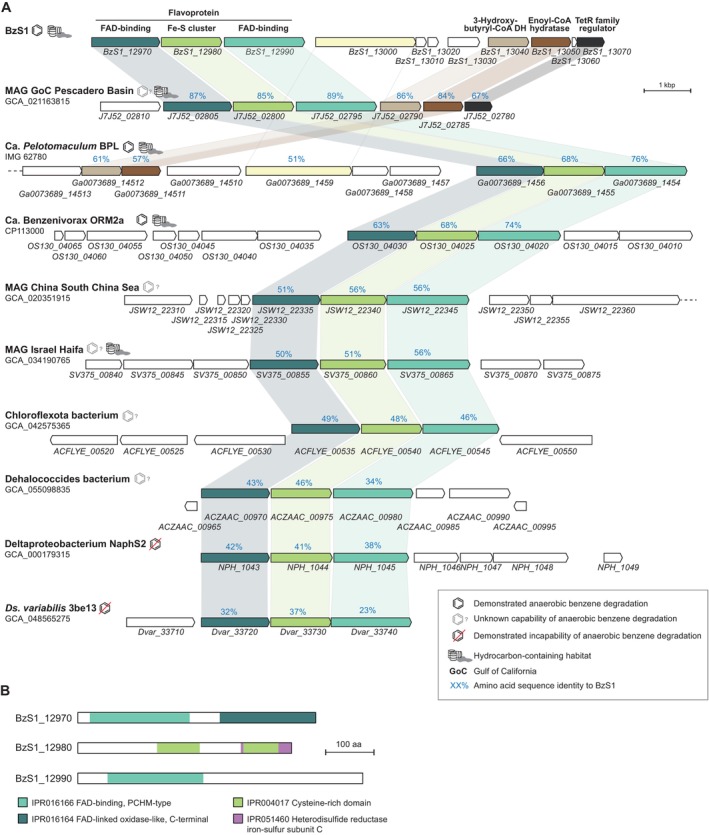
Comparative genomic analyses of the abundant, putatively heterotrimeric flavoprotein from BzS1. Gene cluster (A) and domain structures (B) of the flavoprotein subunits. Accession numbers of genomes from which the shown gene clusters were retrieved are indicated. Further comparisons and geographical origins of compared strains, cultures, and MAGs are presented in Figure S16.

The coding genes *BzS1_12970/80/90* are organised in an operon‐like structure, which is neighboured downstream by four genes (*BzS1_13000/10/20/30*) encoding β‐oxidation‐related enzymes and further by genes for a predicted 3‐hydroxybutyryl‐CoA dehydrogenase (*BzS1_13040*), an enoyl‐CoA hydratase (*BzS1_13050*), and a TetR family regulator (*BzS1_13070*) (Figure 5A, top). The latter affiliates with regulators implicated in transcriptional regulation of gene clusters for anaerobic toluene and *p*‐cresol degradation in, for example, *Db. toluolica* Tol2^T^ (Figure S15) (Wöhlbrand et al. 2013). This regulator might be responsive next to benzene also to toluene, which would explain the similar abundance of the BzS1_12970/80/90 proteins also in toluene‐adapted BzS1 cultures (see Section 3.5.1).

Homologues of the BzS1_12970/80/90 and BzS1_13040/50/70 proteins with high sequence identities (84%–89%) are encoded in a similarly organised operon‐like gene cluster of a MAG (acc. no. GCA_021163815; here termed MAG GoC Pescadero Basin (Speth et al. 2022)) (Figure 5A) originating from hydrocarbon‐rich hydrothermal sediment in the Pescadero Basin of the Gulf of California (GoC). This prolific hydrocarbon basin is rich in diverse hydrocarbon‐degrading microorganisms including sulphate reducers (Simoneit and Lonsdale 1982; Edgcomb et al. 2022). Notably, the *BzS1_12970/80/90* genes are also present in the same arrangement (syntenic) albeit with somewhat lower sequence identities (63%–76%) in the MAGs of *Pelotomaculum* ca. BPL (Dong et al. 2017) and *Ca*. Anaerobenzenivorax ORM2a (Toth et al. 2026), and their protein products were also identified in the respecting benzene‐degrading enrichment cultures. Further syntenic homologues are present in various other MAGs from different geographical locations (Figure 5A and Figure S16). Syntenic homologues with markedly lower sequence identities (23%–42%) are encoded in the genomes of sulphate‐reducing, naphthalene‐degrading strain NaphS2 and versatile *Ds. variabilis*. Considering that strain NaphS2 cannot utilise benzene (Galushko et al. 1999), but 2‐methylnaphthalene and toluene via the respective arylsuccinate synthases Nms and Bss (Musat et al. 2009; DiDonato Jr et al. 2010), and that *Ds. variabilis* also cannot utilise benzene (this study), these homologues likely serve other metabolic functions than being involved in anaerobic degradation of benzene.

While the BzS1_12970 protein of BzS1 harbours an N‐terminal FAD‐binding domain related to *p*‐cresol methylhydroxylase (PCMH) as well as a C‐terminal FAD‐linked oxidase domain, the related BzS1_12990 protein shares only the N‐terminal FAD‐binding domain of the PCMH‐type. The BzS1_12980 protein possesses two cysteine‐rich domains, with the N‐terminal one correlating with an Fe/S cluster (Figure 5B).

#### 3.5.3 Phylogenetic Relations With Other Flavoproteins

**FIGURE 6:**
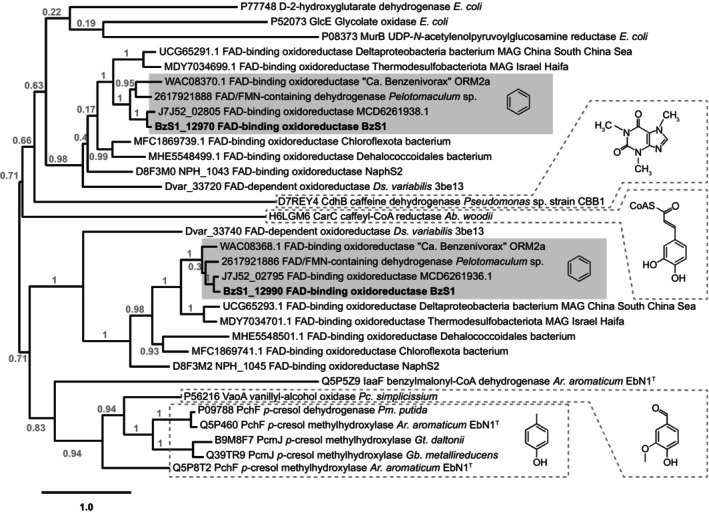
Phylogenetic tree of the two FAD‐binding subunits of the putatively heterotrimeric flavoprotein from BzS1. They tightly cluster with the orthologs from the MAGs from the Gulf of California, *Pelotomaculum* ca. BPL, and *Ca*. Anaerobenzenivorax ORM2 (marked by grey background). Accession numbers of protein sequences are indicated. Detailed phylogenetic trees for each subunit of the putatively heterotrimeric flavoprotein comprising the top 100 BLAST hits are provided in Figures S17–S19.

Phylogenetic analysis revealed that each of the two predicted flavoproteins (BzS1_12970/90) identified in BzS1 forms a coherent cluster with the respective orthologs from the MAG GoC Pescadero Basin, *Pelotomaculum* ca. BPL, and *Ca*. Anaerobenzenivorax ORM2. Each of these two ‘core’ clusters is closely linked to another one representing MAGs from the South China Sea and Haifa (Israel) and somewhat more separated from a cluster containing, among others, the orthologs from strain NaphS2 and *Ds. variabilis* 3be13 (Figure 6). Detailed phylogenetic trees of the subunits constituting the presumptively heterotrimeric flavoprotein of BzS1 are provided in Figures S17–S19. Interestingly, the BzS1_12970‐specific phylogenetic cluster is related to flavin‐binding subunits of membrane‐localised glycolate oxidase (GlcDEF) from *Escherichia coli* that oxidises glycolate to glyoxylate (Lord 1972; Sallal and Nimer 1989; Pellicer et al. 1996) and caffeine dehydrogenase (CdhABC) of gammaproteobacterial *Pseudomonas* sp. strain CBB1 that oxidises the C8 position of caffeine, forming trimethyluricate (Yu et al. 2008; Mohanty et al. 2012). These two heterotrimeric enzymes differ in subunit composition: while glycolate oxidase has two flavin‐binding subunits (GlcDE) and one FeS protein (GlcF) similar to BzS1_12970/80/90, the caffeine dehydrogenase is composed of a molybdopterin‐ (CdhA), a FAD‐ (CdhB), and a [2Fe‐2S]‐binding (ChdC) subunit.

The juxtaposed clusters of BzS1_12970 and BZS1_12990 separate from a heterogeneous cluster harbouring structurally and functionally diverse members of the vanillyl alcohol oxidase/*p*‐cresol methyl hydroxylase (VAO/PCMH) flavoprotein family (Ewing et al. 2017), as exemplified in the following: (i) The UDP‐*N*‐acetylenoylpyruvoylglucosamine reductase (MurB) from *E. coli* employs its flavin cofactor to transfer a hydride from NADPH to enoylpyruvyl‐UDP‐*N*‐acetylglucosamine giving UDP‐*N*‐acetylmuramic acid for peptidoglycan synthesis (Benson et al. 1996). (ii) The electron bifurcating caffeyl‐CoA reductase CarCDE of the firmicute *Acetobacterium woodii* couples reduction of caffeyl‐CoA to dihydrocaffeyl‐CoA with Na^+^‐pumping via the transmembrane RNF complex (Bertsch et al. 2013) and forms a heterododecameric Car(CDE)_4_ complex (Demmer et al. 2018). (iii) The homooctameric vanillyl‐alcohol oxidase (VaoA) from the ascomycete *Penicillium simplicissimum* oxidises vanillyl‐alcohol to vanillin by initial direct hydride transfer from the substrate to the flavin cofactor (Mattevi et al. 1997; Benen et al. 1998). VaoA affiliates with (iv) a subcluster of the flavin‐containing (PchF) subunits of PCMH from various facultative anaerobic microorganisms. PCMH from *Pseudomonas putida* has a heterotetrameric structure and abstracts a hydride from the methyl group of *p*‐cresol (Hopper and Taylor 1977; Cunane et al. 2000, 2005). We refrained from structural predictions of the flavoprotein due to the lack of closely related enzymes with available high‐resolution structures.

## 4 Discussion

### 4.1 Evaluating Previous Hypotheses on Initial Reaction

#### 4.1.1 Carboxylation and Hydroxylation

The sulphate‐reducing enrichment culture N47 is proposed to anaerobically convert naphthalene to 2‐naphthoate, with the involved naphthalene carboxylase employing a prenylated flavin mononucleotide (preFMN) cofactor (Figure S20A) (Heker et al. 2023). Carboxylation was also proposed for anaerobic benzene degradation by the iron‐reducing enrichment culture BF (Abu Laban et al. 2010), the hyperthermophilic archaeon *Ferroglobus placidus* (Holmes et al. 2011), a nitrate‐reducing continuous culture (Atashgahi et al. 2018), and *Peptococcaceae* spp. in a nitrate‐reducing, syntrophic enrichment culture (Luo et al. 2014). Originally, prFMN‐dependent Fdc1 (de)carboxylase from the common mould *Aspergillus niger* was shown to reversibly carboxylate styrene to *trans*‐cinnamate (Payne et al. 2015). Such a reaction is apparently not used by BzS1 nor by naphthalene‐degrading strain NaphS2, since their genomes do not harbour genes for a respective prFMN‐dependent carboxylase, as was previously also reported for *Pelotomaculum* ca. BPL (Dong et al. 2017) and most recently for *Ca*. Anaerobenzenivorax ORM2 (Toth et al. 2026).

Carboxylation in analogy to anaerobic phenol degradation (Figure S20B) also appears unlikely in the case of BzS1, since no genes for a phenylphosphate carboxylase PpcABC (Schühle and Fuchs 2004) and preceding ATP‐dependent phenylphosphate synthase (Narmandakh et al. 2006) could be predicted in its genome. A preceding hydroxylation of benzene to phenol also appears unlikely, since dark intracellular O_2_‐generation fuelling a required monooxygenase (Ettwig et al. 2012) can be excluded. Finally, early observations indicating phenol as an intermediate of anaerobic benzene degradation (Grbić‐Galić and Vogel 1987) may have been compromised by an abiotic formation upon retrieval and processing of culture samples (Kunapuli et al. 2008).

Our labelling experiments with culture BzS1 applying (^13^C_6_)benzene demonstrated that the carboxyl carbon atom of detected benzoate is predominantly benzene‐derived (Figure 2C). Furthermore, genome analysis revealed the absence of a gene encoding a benzoate‐CoA ligase, which would be required to feed a benzoate intermediate into the central benzoyl‐CoA pathway. This corroborates earlier findings with sulphate‐reducing/sulphidogenic consortia showing that the carboxyl group of benzoate originates from supplied (^13^C_6_)benzene (Caldwell and Suflita 2000) and that the label of applied H^13^CO_3_^−^ could not be recovered in the carboxyl group (Phelps et al. 2001). In the context of the latter, it is noteworthy that a bicarbonate uptake system is apparently not encoded in the BzS1 genome. Taken together, several lines of evidence give reason to doubt benzene activation by a direct carboxylation to benzoate in BzS1.

#### 4.1.2 Methylation

Previously, methylation of benzene by an unknown methyl donor giving toluene was hypothesised as the initial transformation reaction (Figure S20C) (Coates et al. 2002). Formed toluene would then be converted to benzoyl‐CoA via the well‐known pathway (see Section 3.4), making a benzoate‐CoA ligase unnecessary. This reaction sequence was more recently also suggested for the deltaproteobacterial *Geotalea daltonii* FRC‐32^T^ (Bullows et al. 2024), which, however, appears unlikely since the catalytically implicated hydroxybenzylsuccinate synthase (HbsA) (i) affiliates with toluene‐activating arylalkylsuccinate synthases (Figure S21) and (ii) other HbsAs subunits related to activation of *p*‐cresol to (4‐hydroxybenzyl)succinate form a clearly separated phylogenetic branch (Wöhlbrand et al. 2013). Finally, also metagenomics‐inferred exclusion of methylation was previously reported for *Pelotomaculum* ca. BPL (Dong et al. 2017) and *Ca*. Anaerobenzenivorax ORM2 (Toth et al. 2026).

In the case of culture BzS1, incubation experiments with a mixture of (^13^C_6_)benzene and unlabelled toluene yielded unlabelled or (^13^C_1–4_)‐side chain labelled benzylsuccinate, but no ring‐labelled product (Figure 1 and Figure S7C). Furthermore, the products of the *bss* and *bbs* operons were undetectable in benzene‐adapted BzS1 cultures, while the vast majority of them were detected at high abundance in toluene‐adapted cultures (Figure 4B). Lastly, membrane‐permeable toluene supported growth only after a long lag phase, while rapid utilisation would have been expected for a true intermediate. Taken together, our current data are at odds with benzene activation via initial methylation to toluene in BzS1.

### 4.2 Considerations on Alternative Cosubstrates for Initial Reaction

Given that the common hypotheses on the initial reaction of anaerobic benzene degradation, that is, carboxylation, hydroxylation, and methylation (see Sections 4.1.1 and 4.1.2), proved as not applicable for BzS1, we considered other mechanistically plausible C_1_–C_3_ cosubstrates that would allow conversion of benzene to benzoyl‐CoA without intermediate formation of benzoate and, apart from that, be also consistent with our metabolite and proteogenomic data.

#### 4.2.1 Carboxyphosphate

Using carboxyphosphate for an electrophilic substitution of benzene would generate benzoyl‐phosphate, which could be easily converted to benzoyl‐CoA by a CoA‐transferase (Figure S20D). This would waive the need for (exogenous) CO_2_ and agree with the observed retaining of the ^13^C‐label. Two possibilities for the provisioning of carboxyphosphate are conceivable: (i) Carboxyphosphate can be formed by a reversely operating carbamoylphosphate synthase (Thoden et al. 2002), which is predicted but detected only at low levels in BzS1 cultures, or by a biotin carboxylase (Chou et al. 2009), which is, however, not predicted. (ii) Alternatively, an enzyme that functions like heterotrimeric, membrane‐localised glutaconyl‐CoA decarboxylase (Gdc) could be involved: GdcA cleaves the C—COO^−^ bond and transfers the carboxyl group to biotin‐containing GdcC, which is decarboxylated by GdcB (Wendt et al. 2003). The genome of BzS1 harbours potential *gdcA*, *B* and *C* genes, with putative GcdA2 (BzS1_25310) representing a presumptively fused trans‐/decarboxylase‐carbamoylphosphate‐synthetase. This could transform a glutaconyl‐CoA derived carboxyl group to an enzyme bound carboxyphosphate (Figure S20E). While such a reaction would be biochemically appealing, the detection of GcdA2 only at very low abundance in BzS1 cultures renders its involvement in such a metabolic key reaction questionable at present.

#### 4.2.2 Acetyl‐CoA

Formation of phenylacetyl‐CoA from benzene and acetyl‐CoA, while difficult to imagine as it would require carbon bond formation between two nucleophilic carbon atoms, would allow direct entry into the known anaerobic phenylacetyl‐CoA pathway. However, our experiments with 1/2‐(^13^C)acetate suggest that the provided acetate is not a relevant source for the carboxyl group of the detected benzoate. A similar observation was previously reported by Phelps et al. (2001). Furthermore, from a genomic perspective, phenylacetate (respectively its CoA activated form) does not appear as a likely intermediate (Figure S20F), since no clear candidate genes coding for pathway‐relevant enzymes could be predicted in the BzS1 genome, that is, phenylacetyl‐CoA dehydrogenase, thioesterase, and NADH‐dependent phenylglyoxylate dehydrogenase (e.g., Rhee and Fuchs 1999).

So far, our data point to benzene degradation by BzS1 via benzoyl‐CoA, with its carbonyl group originating from a multi‐carbon compound. The latter in turn should be formed during benzoyl‐CoA degradation or deriving from it, and be reactive for adding to an activated benzene. We are considering the following possibilities, as outlined in the two sections below.

#### 4.2.3 Pyruvate, Phosphoenolpyruvate or Acrylyl‐CoA

**FIGURE 7:**
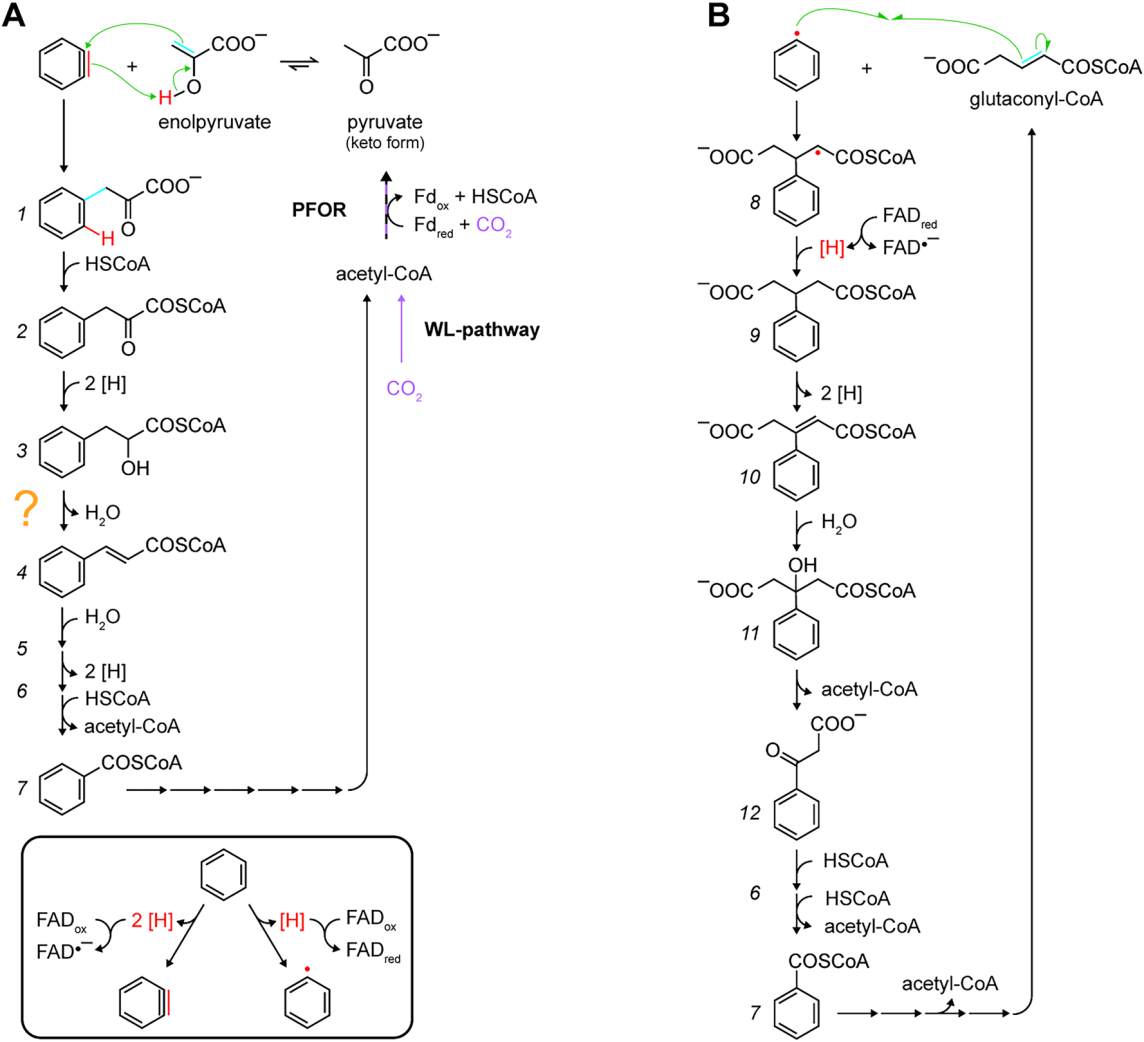
Hypothesised role of the presumptively heterotrimeric flavoprotein (BZS1_12970/80/90) in anaerobic benzene activation and subsequent reaction sequences giving benzoyl‐CoA in BzS1. (A) Nucleophilic substitution of benzyne with enolpyruvate (via cyclic intermediate, not shown) versus (B) addition of a phenyl radical to glutaconyl‐CoA. Compound names: (1), 2‐oxo‐3‐phenylpropanoate; (2), 2‐oxo‐3‐phenylpropanoyl‐CoA; (3), 2‐hydroxy‐3‐phenylpropanoyl‐CoA; (4), (2*E*)‐3‐phenylprop‐2‐enoyl‐CoA (synonym: cinnamoyl‐CoA); (5), 3‐hydroxy‐3‐phenylpropanoyl‐CoA; (6), 3‐oxo‐3‐phenylpropanoyl‐CoA; (7), benzoyl‐CoA; (8), 3‐phenylglutaryl‐CoA radical; (9), 3‐phenylglutaryl‐CoA; (10), 3‐phenylglutaconyl‐CoA; (11), 3‐hydroxy‐3‐phenylglutaryl‐CoA; (12), 3‐oxo‐3‐phenylpropanoate; ‘?’, α‐elimination of water by a yet unassigned dehydratase; additional information is provided in Figure S23. Box: Possible initial activation of benzene by C—H bond cleavage: Abstraction of either two electrons and two protons giving a benzyne (left) or of one electron and one proton generating a phenyl radical (right).

Considering the detection of 3‐phenylpropanoate in substantial quantities under essentially all studied cultivation conditions (Figure 1) lets us assume that benzene might be added terminally to the C=C bond of a C_3_‐metabolite, such as enolpyruvate and its energy‐rich derivative phosphoenolpyruvate (PEP), or acrylyl‐CoA. The functional groups at C2 of enolpyruvate and PEP promote the nucleophilic character of the C3 atom, while in acrylyl‐CoA the C3 atom has low electron density and thus is unlikely to act as a nucleophile (Figure S20A). For benzene to undergo nucleophilic substitution with the respective C=C bond of enolpyruvate or PEP, its prior oxidation to benzyne (C_6_H_4_) is required (Figure 7, box). Such an initial reaction is appealing since it would reconcile several of our observations not coherent at first glance: (i) possibility of an aryne mechanism as indicated by the experiments with fluorobenzene, (ii) consistent detection of 3‐phenylpropanoate and, at lower abundance, phenylacetate, as well as (iii) the non‐uniform labelling patterns in the C_2_/C_3_ side chains of phenylacetate/‐propanoate. Notably, previous compound‐specific stable isotope analyses have revealed carbon and hydrogen atoms to be involved in the rate‐limiting bond‐breaking step of the initial reaction of non‐substituted aromatic hydrocarbon degradation by sulphate‐reducing bacteria (Bergmann et al. 2011), which would be in agreement with the initial formation of benzyne.

BzS1 could synthesise pyruvate and PEP with different degrees of ^13^C‐labelling via the following partly connected metabolic modules: (i) (^13^C_2_)acetyl‐CoA (and ^13^CO_2_) is formed by degradation of benzoyl‐CoA derived from (^13^C_6_)benzene, while (^13^C_0–2_)acetyl‐CoA can be generated from ^12/13^CO_2_ by the WL‐pathway operating in reverse (reductive) direction (Länge et al. 1989; Schauder et al. 1989). (ii) Likewise reductively operating PFOR then forms (^13^C_0–3_)pyruvate from (^13^C_0–2_)acetyl‐CoA and ^12/13^CO_2_ (Jansen et al. 1984; Furdui and Ragsdale 2000). (iii) PEP synthetase (PpsA) finally transforms (^13^C_0–3_)pyruvate to (^13^C_0–3_)PEP. Pyruvate reduction by lactate dehydrogenase and activation with coenzyme A would generate lactyl‐CoA, which can be converted to acrylyl‐CoA by lactyl‐CoA dehydratase (Kuchta and Abeles 1985; Hofmeister and Buckel 1992).

Addition of benzyne to enolpyruvate or PEP (hydrolysis of phosphoryl ester (−61 kJ/mol) could energise the reaction) would give 2‐oxo‐3‐phenylpropanoate (Figure 7A). The latter could be converted to (2*E*)‐3‐phenylprop‐2‐enoyl‐CoA (synonym: cinnamoyl‐CoA). This would, however, require the activity of a phenyllactyl‐CoA dehydratase as known from anaerobically L‐phenylalanine‐degrading *Clostridium sporogenes* (Dickert et al. 2002). Subsequent conversion of cinnamoyl‐CoA to 3‐oxo‐3‐phenylpropanoyl‐CoA would allow its thiolytic cleavage into benzoyl‐CoA and acetyl‐CoA. Non‐productive side reactions acting on cinnamoyl‐CoA could give observed 3‐phenylpropanoate. The involved β‐oxidation like reactions transforming 2‐oxo‐3‐phenylpropanoate into benzoyl‐CoA could be carried out by the suitable enzymes encoded directly downstream of the *BzS1_12970/80/90* genes (see Figure 5A, top). Such an initial benzene transformation reaction may also explain the formation of phenylacetate as a putative dead‐end product by (abiotic) oxidative decarboxylation of 2‐oxo‐3‐phenylpropanoate. This would be in analogy to other α‐oxo acids known to rapidly oxidatively decarboxylate by various mild oxidants (Hanson 1987). Notably, it was recently demonstrated that protometabolic reduction of NAD^+^ under anoxic conditions may be coupled to oxidative decarboxylation of α‐oxo acids (Basak et al. 2021).

Addition of benzene to acrylyl‐CoA would proceed via an electrophilic aromatic substitution directly giving 3‐phenylpropanoyl‐CoA. Subsequent dehydrogenation to cinnamoyl‐CoA followed by a single round of β‐oxidation could on the shortest route yield benzoyl‐CoA and acetyl‐CoA. In such a reaction, the C3 of acrylyl‐CoA would attack a carbon atom of benzene resulting in a σ‐complex from which a proton would be released. While this is an attractive and simple pathway, it has the drawback that a significant [H]‐exchange as observed with (^2^H_6_)benzene (see Section 3.2.3) would not be expected. Alternatively, one may consider addition of a phenyl radical (C_6_H_5_^•^) in analogy to the reaction with glutaconyl‐CoA (see Section 4.2.4).

To further assess the suggested dehydratase reaction (cinnamoyl‐CoA‐forming), we inspected the proteogenomic data of BzS1. This provided hints on favouring the presence of a phenyllactyl‐CoA dehydratase over that of a lactyl‐CoA dehydratase: (i) All products of the gene cluster *BzS1_26180/190/200/210* were detected at high abundance (consistently higher in benzene‐ than in toluene‐adapted cultures). The BzS1_26180/90 proteins are related to cinnamoyl‐CoA:phenyllactate CoA‐transferase, which represents a subunit of heterotrimeric (FldABC) phenyllactyl‐CoA dehydratase from *Cl. sporogenes* (Dickert et al. 2002). Furthermore, the BzS1_26200 protein harbours a dehydratase domain, while the BzS1_26210 protein represents a likely acyl‐CoA synthetase (Figure S23). (ii) The singular gene *BzS1_13720* encodes a protein (not detected) with fair sequence identities (30%) to the catalytic FldB subunit of phenyllactyl‐CoA dehydratase. (iii) The gene cluster *BzS1_02010–60* codes for proteins (not detected) with mediocre sequence identities (~26%) to subunits of 2‐hydroxyglutaryl‐CoA dehydratase from *Cl. symbiosum* (Parthasarathy et al. 2011). Thus, BzS1 could indeed have the capacity for α‐elimination of water.

#### 4.2.4 Glutaconyl‐CoA

The central anaerobic benzoyl‐CoA pathway generates the open chain intermediate glutaryl‐CoA, which strict anaerobes then dehydrogenate to glutaconyl‐CoA using a non‐decarboxylating, FAD‐dependent glutaryl‐CoA dehydrogenase (GDH) (Wischgoll et al. 2010).

Assuming that glutaconyl‐CoA might be the cosubstrate of the initial reaction with benzene, as most recently also proposed by Toth et al. (2026), an addition of a phenyl radical (C_6_H_5_^•^) (Figure 7, box) to the double bond of glutaconyl‐CoA, forming—via a radical intermediate—3‐phenylglutaryl‐CoA, appears feasible (Figure 7B). Subsequent reactions would lead to benzoyl‐CoA and two acetyl‐CoA using conventional steps and enzymes. Genes encoding some of the implied enzymes, that is, enoyl‐CoA hydratase/isomerase and 3‐hydroxyacyl‐CoA dehydrogenase, are present in the benzene‐induced gene cluster (Figure 5A, top). As for benzyne (see Section 4.2.3), the formation of a phenyl radical requires C—H bond cleavage, which is also in agreement with the known isotope fractionation (Bergmann et al. 2011).

While the initial formation of 3‐phenylglutaryl‐CoA would be reminiscent of benzyl/(methylalkyl)succinate formation in anaerobic degradation of toluene/*n*‐alkanes, radical generation and cosubstrate would differ markedly (Figure 7B). Previous labelling experiments with *Aromatoleum* sp. strain HxN1 suggested a concerted mechanism for radical addition of *n*‐hexane to fumarate avoiding the free subterminal alkyl radical intermediate (Jarling et al. 2012). Considering that the phenyl radical is even more reactive than a secondary alkyl radical, such a scenario should also apply to a reaction involving a phenyl radical. We assume that [H]‐abstraction and addition of the phenyl ring to glutaconyl‐CoA would thus occur instantaneously. This would not allow for any [H]‐exchanging processes that could explain significant loss of ^2^H atoms (see Section 3.2.3) when (^2^H_6_)benzene is provided. Furthermore, the incomplete isotope labelling in the C_3_ side chain of 3‐phenylpropanoate detected with (^13^C_6_)benzene (see Section 3.2.1) questions that this side chain originates from a glutaconyl‐CoA derived directly from benzene. Thus, the speculated ‘glutaconyl‐CoA’ pathway should be viewed with some prudence at this time.

Alternatively, benzene might be added to the CoA‐activated group of glutaconyl‐CoA, giving phenylglutaconate (5‐phenyl‐5‐oxopent‐3‐enoate). Its conversion to benzoyl‐CoA and two acetyl‐CoA would require only four additional enzymes, that is, CoA ligase or CoA transferase, 2,3‐enoyl‐CoA isomerase, enoyl‐CoA hydratase, 3‐hydroxyacyl‐CoA dehydrogenase, and thiolase, which may be recruited from predicted β‐oxidation related enzymes.

### 4.3 How the Abundant Flavoprotein Could Be Involved in Initial Reaction

The conspicuous domain architectures of the three dominant proteins of BzS1, which are also conserved in *Pelotomaculum* ca. BPL (Dong et al. 2017) and *Ca*. Anaerobenzenivorax ORM2 (Toth et al. 2026), namely two FAD‐binding domains related to *p*‐cresol methylhydroxylase PCMH (in BzS1_12970/90), a further FAD‐binding domain in BzS1_12970, and a FeS‐like domain (in BzS1_12980), prompt comparison to PCMH as well as to flavin‐based electron bifurcation/confurcation (FBEB/FBEC) as outlined in the following:

#### 4.3.1 Analogy of BzS1_12970/80/90 to PCMH

PCMH has an α_2_β_2_‐heterotetrameric structure, with the large α‐subunits (PchFs) containing each an FAD cofactor and the small β‐subunits (PchCs) representing *c*‐type cytochromes. During catalysis, the FAD cofactor receives 2 electrons from the oxidation of the benzylic methyl group of *p*‐cresol yielding a quinone‐methide intermediate. While the reduced FAD passes on the electron equivalents to the heme cofactor, the methylene (=CH_2_) group of the intermediate reacts with water to a primary alcohol, forming the product *p*‐hydroxybenzyl alcohol (Figure S20H) (Cunane et al. 2000, 2005).

It should be noted that the presence of the *p*‐hydroxyl group in *p*‐cresol is mandatory for formation of the resonance‐stabilised quinone‐methide intermediate, a scenario not applicable to the unsubstituted benzene molecule. Thus, if the FAD‐binding proteins BzS1_12970/90 were also to function in oxidative direction, a benzyne (C_6_H_4_) would have to be generated, as required for nucleophilic aromatic substitution, with released electrons drained off via the FeS protein BzS1_12980. How the two FAD‐binding proteins BzS1_12970/90 should interact/cooperate remains unclear at present. Such a reaction type would agree with an aryne mechanism indicated by our labelling experiment with fluorobenzene and be novel to biocatalysis, or simply not be feasible.

#### 4.3.2 Analogy of BzS1_12970/80/90 to FBEB/FBEC

FBEB, which occurs in strictly anaerobic prokaryotes, couples the sequential two‐electron reduction/reoxidation of flavin to the one‐electron reduction of a high‐ and a low‐potential acceptor, a process called electron bifurcation (Buckel and Thauer 2018; Kayastha et al. 2021). A well‐understood example is the bifurcating electron transferring flavoprotein/butyryl‐CoA dehydrogenase ((EtfAB‐Bcd)_4_) complex used by *Clostridium difficile* during butyrate fermentation (Demmer et al. 2017). Essentially, NADH reduces the β‐FAD (to β‐FADH^−^) in EtfB, which bifurcates by reducing a ferredoxin (uphill) and the α‐FAD^•−^ (to α‐FADH−) in domain II of EtfA (downhill). The latter swings to the δ‐FAD in Bcd and reduces it to δ‐FADH•, which is then used to reduce ½ crotonyl‐CoA to ½ butyryl‐CoA (two rounds required for complete reduction) (Figure S22B). Extending the concept of FBEB, recently also the opposite electron flow was demonstrated for the lactate‐oxidising (to pyruvate) lactate dehydrogenase—electron transferring flavoprotein ((Ldh‐EtfAB)_2_) complex of *Ac. woodii* (Kayastha et al. 2022), which integrates FBEB and FBEC. Essentially, electrons from Ldh‐localised δ‐FADH^−^ are bifurcated to a low potential ferredoxin and a higher potential α‐FAD (generating α‐FAD^•−^/α‐FADH^−^ in EtfA), followed by confurcation of electrons in EtfB to give β‐FADH^−^, which finally transfers a hydride to NAD^+^ (Figure S22C).

Assuming that the BzS1_12970/80/90 proteins form a heterotrimeric flavoprotein that operates in analogy to FBEB/FBEC (as also indicated for *Ca*. Anaerobenzenivorax ORM2 (Toth et al. 2026)), one may speculate that the BzS1_12990 subunit with the single FAD‐binding motif would be involved in generating benzyne (or phenyl radical) by bifurcating (two versus one half cycle) the released electron(s) to ferredoxin and the BzS1_12970 subunit with the two FAD‐binding motifs. The latter could then conduct the electron confurcation, possibly to the HdrC‐like subunit (BzS1_12980). The high abundance of the electron transfer protein EtfA3B3 (distinct from the one associated with anaerobic toluene degradation) in benzene‐utilising BzS1 cultures and the presence of highly similar and syntenic orthologs in the MAG GoC Pescadero Basin (Figure S24) indicates a possible involvement in further electron transfer from the flavoprotein.

## 5 Conclusions and Outlook

The long‐known main reaction principle used by aerobic bacteria to initiate degradation of hydrocarbons is based on the universal O_2_‐dependence of mechanistically diverse mono‐ and dioxygenases to introduce hydroxyl groups into aromatic rings. By contrast, naphthalene carboxylase employing a prenylated flavin mononucleotide cofactor is so far the only mechanistically studied mode of anaerobic transformation of an unsubstituted aromatic hydrocarbon. The here reported initial evidence for benzene activation to possibly involve a novel type of flavoenzyme represents another potential strategy for anaerobic activation of such hydrocarbons. This assumption is substantiated by the most recent analogous findings with a methanogenic, benzene‐degrading enrichment culture (Toth et al. 2026) and various MAGs from other hydrocarbon‐rich habitats at geographically distant locations (see Figure 5 and Figure S16B). Therefore, research with culture BzS1 (and other available cultures) should be realigned with a series of newly emerging key questions. To address them will require studies with the (heterologous or native) purified multimeric enzyme (BzS1_12970/80/90, putative flavin‐dependent benzene dehydrogenase (BeDH)) and potentially associated electron‐transferring proteins (e.g., EtfA3B3) of BzS1. Of foremost concern should be the mode of initial benzene activation (benzyne or phenyl radical), the actual cosubstrate (e.g., enolpyruvate, PEP, or glutaconyl‐CoA) utilised in the initial reaction as well as the subsequent reactions generating benzoyl‐CoA. Considering the high abundance of the flavoprotein in BzS1, structural investigations towards an in‐depth mechanistic understanding might be feasible even based on native purification, as recently shown for methyl‐coenzyme M reductase (MCR) from anaerobic methanotrophic archaea (ANME) (Müller et al. 2025). Furthermore, it will be interesting to learn about the genetic dissemination of these flavoproteins and associated β‐oxidation enzymes across different anaerobic respiration types and the biogeography of MAGs obtained from hydrocarbon/benzene‐containing (marine) habitats.

## Author Contributions

**Florin Musat:** conceptualization, investigation, writing – review and editing, methodology. **Lars Wöhlbrand:** investigation, visualization, writing – review and editing, methodology. **Rene Jarling:** investigation, methodology. **Niculina Musat:** investigation, methodology. **Rudolf Amann:** funding acquisition. **Richard Reinhardt:** investigation, resources. **Stefan Bruns:** investigation. **Michael Kube:** investigation. **Friedrich Widdel:** conceptualization, writing – review and editing, resources. **Patrick Becker:** investigation, visualization. **Heinz Wilkes:** investigation, writing – review and editing, methodology. **Ralf Rabus:** investigation, writing – review and editing, writing – original draft, visualization.

## Funding

This work was supported by Max Planck Gesellschaft.

## Ethics Statement

The authors have nothing to report.

## Conflicts of Interest

The authors declare no conflicts of interest.

## Supporting information


**Table S1:** Oligonucleotide probes used to resolve the microbial community of the benzene‐degrading, sulphate‐reducing enrichment culture and to probe the purity of culture BzS1.
**Figure S1:** Five‐step workflow applied in this study.
**Figure S2:** Characterisation of the benzene‐degrading enrichment culture using isolation, whole‐cell hybridisation, stable isotope labelling, and nanoSIMS analyses.
**Figure S3:** Physiological experiments with benzoate‐degrading strains isolated from the benzene‐degrading, sulphate‐reducing enrichment culture.
**Figure S4:** Physiological experiments with sulphate‐reducing culture BzS1.
**Figure S5:** Mass spectra of benzoates (as methyl esters).
**Figure S6:** Mass spectra of phenylacetates (as methyl esters).
**Figure S7:** Mass spectra of 3‐phenylpropanoates (as methyl esters).
**Figure S8:** Ion chromatograms (*m*/*z* = 154 + 123 + 95) revealing the elution order and relative abundance of the fluorobenzoates (as methyl esters).
**Figure S9:** Ion chromatograms (*m*/*z* = 168 + 109 + 83) revealing the elution order and relative abundance of the fluorophenylacetates (as methyl esters).
**Figure S10:** Ion chromatograms (*m*/*z* = 182 + 123 + 109) revealing the elution order and relative abundance of the 3‐(fluorophenyl)propanoates (as methyl esters).
**Figure S11:** Gene clusters and proteomic detection for selected metabolic processes in BzS1.
**Figure S12:** Coomassie‐stained 2DE‐gel from the benzene‐degrading enrichment culture.
**Figure S13:** Coomassie‐stained 1DE‐gel from the soluble fraction of the benzene‐degrading enrichment culture.
**Figure S14:** Coomassie‐stained SDS‐gel from the membrane protein‐enriched fraction of the benzene‐degrading enrichment culture.
**Figure S15:** Phylogenetic clustering of TetR family regulator (BzS1_13070) from BzS1.
**Figure S16:** Additional comparative analyses of the abundant, putatively heterotrimeric flavoprotein from BzS1.
**Figure S17:** Phylogenetic affiliations of putative flavoprotein subunit BzS1_12970.
**Figure S18:** Phylogenetic affiliations of putative flavoprotein subunit BzS1_12980.
**Figure S19:** Phylogenetic affiliations of putative flavoprotein subunit BzS1_12990.
**Figure S20:** Previously hypothesised and other conceivable reactions for the initial step(s) of anaerobic benzene degradation and further potentially relevant reactions.
**Figure S21:** Phylogenetic relations of catalytic subunits from aryl‐/alkylsuccinate synthases.
**Figure S22:** Additional information on possible role of presumptively heterotrimeric flavoprotein BZS1_12970/80/90.
**Figure S23:** Possible candidate for α‐elimination of water from 2‐hydroxy‐3‐phenylpropanoyl‐CoA.
**Figure S24:** Abundant electron transfer protein EtfA3B3.

**Table S2:** Overall proteomic dataset generated in the present study.

**Table S3:** Proteogenomic data underlying the tailored catabolic network (Figure 4).

**Table S4:** BLAST results (top 100 hits) underlying the phylogenetic trees for the subunits of the putatively heterotrimeric flavoprotein (Figures S17–S19).

## Data Availability

All data needed to evaluate the conclusions in the paper are present in the paper and/or Supporting Information. This Whole Genome Shotgun project has been deposited at DDBJ/ENA/GenBank under the accession JBVNVM000000000. The version described in this paper is version JBVNVM010000000. The mass spectrometry proteomics data has been deposited at the PRIDE database under the accession PXD080844 and at the FAIRDOMHub repository under the link https://doi.org/10.15490/fairdomhub.1.investigation.823.1.
