# Supplementary material for: Putative Mechanisms for Initial Reaction of Anaerobic Benzene Degradation Presumably Involving a Flavoprotein
Source: Environ Microbiol. 2026 Jul 28;28(8):e70394. doi: 10.1111/1462-2920.70394 (PMC13416013; doi:10.1111/1462-2920.70394)
Supplement: Supplementary file 1 — Table S1: Oligonucleotide probes used to resolve the microbial community of the benzene‐degrading, sulphate‐reducing enrichment culture and to probe the purity of culture BzS1. Figure S1: Five‐step workflow applied in this study. Figure S2: Characterisation of the benzene‐degrading enrichment culture using isolation, whole‐cell hybridisation, stable isotope labelling, and nanoSIMS analyses. Figure S3: Physiological experiments with benzoate‐degrading strains isolated from the benzene‐degrading, sulphate‐reducing enrichment culture. Figure S4: Physiological experiments with sulphate‐reducing culture BzS1. Figure S5: Mass spectra of benzoates (as methyl esters). Figure S6: Mass spectra of phenylacetates (as methyl esters). Figure S7: Mass spectra of 3‐phenylpropanoates (as methyl esters). Figure S8: Ion chromatograms (m/z = 154 + 123 + 95) revealing the elution order and relative abundance of the fluorobenzoates (as methyl esters). Figure S9: Ion chromatograms (m/z = 168 + 109 + 83) revealing the elution order and relative abundance of the fluorophenylacetates (as methyl esters). Figure S10: Ion chromatograms (m/z = 182 + 123 + 109) revealing the elution order and relative abundance of the 3‐(fluorophenyl)propanoates (as methyl esters). Figure S11: Gene clusters and proteomic detection for selected metabolic processes in BzS1. Figure S12: Coomassie‐stained 2DE‐gel from the benzene‐degrading enrichment culture. Figure S13: Coomassie‐stained 1DE‐gel from the soluble fraction of the benzene‐degrading enrichment culture. Figure S14: Coomassie‐stained SDS‐gel from the membrane protein‐enriched fraction of the benzene‐degrading enrichment culture. Figure S15: Phylogenetic clustering of TetR family regulator (BzS1_13070) from BzS1. Figure S16: Additional comparative analyses of the abundant, putatively heterotrimeric flavoprotein from BzS1. Figure S17: Phylogenetic affiliations of putative flavoprotein subunit BzS1_12970. Figure S18: Phylogenetic affiliations of putative fla [file EMI-28-e70394-s003.pdf]

Supplementary Material

SUBSTRATES TESTED, BUT NOT UTILISED:

By the enrichment culture: naphthalene, 2-methylnaphthalene, ethylbenzene, acetophenone, mandelate, 3-phenylpropanoate, phenylacetate, *p*-hydroxybenzoate, phenol, benzyl alcohol, benzaldehyde, formate, glutarate, pimelate, fumarate, succinate, glycine, L-serine, L-alanine, and L-phenylalanine.

By strain BzS1: phenol, acetophenone, phenylalanine, *p*-hydroxybenzoate, benzyl alcohol, benzaldehyde, cyclohexanecarboxylate, fumarate, succinate, lactate, formate, H_2+CO_2, and L-alanine

TABLE S1 | Oligonucleotide probes used to resolve the microbial community of the benzene-degrading, sulphate-reducing enrichment culture and to probe the purity of strain BzS1.

Probe name	Probe sequence	FA (%)	Target	References
DTIG143	5’-TTCGAAGGGTTATCCCGG-3’	20	*Desulfotignum* clade, related phylotypes of the BznS295 enrichment culture[1]	This study
BZN649	5’-CCCCTCCCATACTCAAGT-3’	50	Strain BzS1, specific probe	Musat and Widdel 2008
BZN66	5’-CAAGCCCAGGGTTTCTCG-3’	20	Strain BzS1, specific probe	Musat and Widdel 2008
DSS658	5’-TCCACTTCCCTCTCCCAT-3’	60	*Desulfosarcina* clade, related phylotypes of the BznS295 enrichment culture[1]	Manz et al. 1998
DSB985	5’-CACAGGATGTCAAACCCAG-3’	20	*Desulfobacteraceae* clade, related phylotypes of the BznS295 enrichment culture[a]	Manz et al. 1998
EUB338I-III	5’-GCTGCCTCCCGTAGGAGT-3’<br>5’-GCAGCCACCCGTAGGTGT-3’<br>5’-GCTGCCACCCGTAGGTGT-3’	35	Strain BzS1, all other Bacteria	Amann et al.<br>Daims et al. 1999
NON338	5’-ACTCCTACGGGAGGCAGC-3’	35	None	Wallner et al. 1993

FA: formamide concentration (% v/v) used in FISH.

[a] See reference Musat and Widdel (2008), Supplementary Information and Figure S1.

References

Amann RI, Krumholz L, Stahl DA (1990) Fluorescent-oligonucleotide probing of whole cells for determinative, phylogenetic, and environmental studies in microbiology. J Bacteriol 172:762–770.

Daims H, Brühl A, Amann R, Schleifer K-H, Wagner M (1999) The domain-specific probe EUB338 is insufficient for the detection of all bacteria: development and evaluation of a more comprehensive probe set. Syst Appl Microbiol 22:434–444.

Manz W, Eisenbrecher M, Neu TR, Szewzyk U (1998) Abundance and spatial organization of Gram-negative sulfate-reducing bacteria in activated sludge investigated by in situ probing with specific 16S rRNA targeted oligonucleotides. FEMS Microbiol Ecol 25:43–61.

Musat F, Widdel F (2008) Anaerobic degradation of benzene by a marine sulfate-reducing enrichment culture, and cell hybridization of the dominant phylotype. Environ Microbiol 10:10–19.

Wallner G, Amann R, Beisker W (1993) Optimizing fluorescent in situ hybridization with rRNA-targeted oligonucleotide probes for flow cytometric identification of microorganisms. Cytometry 14:136–143

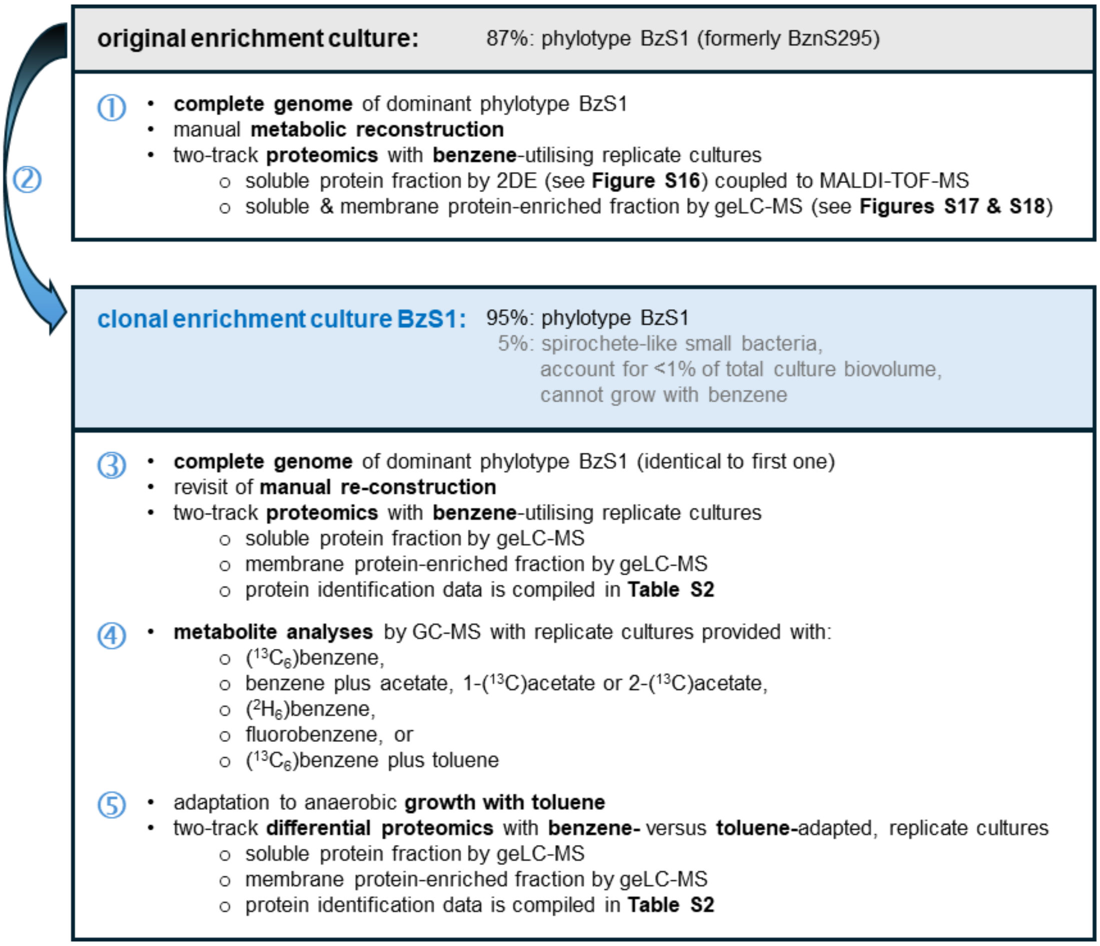

FIGURE S1 | Scheme of our five-step workflow, with its order also mirroring the time course of investigation. The original enrichment culture reflects the one previously reported by Musat and Widdel (2008) and represents the starting point of the present study. Supplementary data on proteomic identification are provided as indicated.

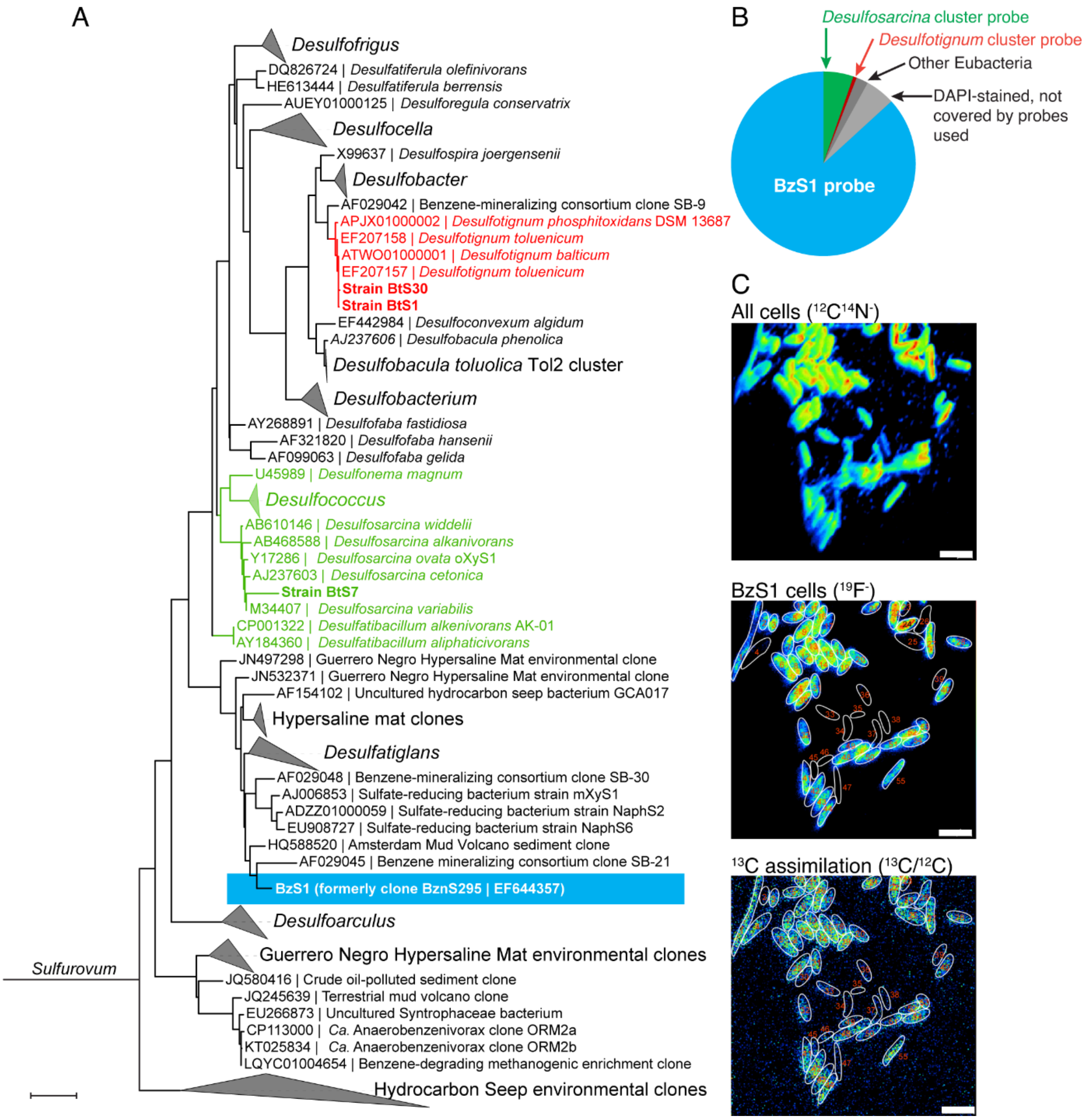

FIGURE S2 | Characterisation of the benzene-degrading enrichment culture using isolation, whole-cell hybridisation, stable isotope labelling and nanoSIMS analyses. **A**. Phylogenetic affiliation of the strains isolated from the clonal enrichment culture using benzoate as a substrate (marked in boldface red and green); benzene-degrading clone BzS1is marked with blue background coloring. Scale bar represent 10% sequence divergence. These strains did not utilized benzene, as demonstrated by growth experiments *(Supplementary above)*. **B**. The structure of the enrichment culture as revealed by whole-cell hybridisations with sequence- and group-specific oligonucleotide probes. Strains and phylotypes targeted by the oligonucleotide probes used are indicated by matching colors in the phylogenetic tree. **C**. HISH-SIMS analysis of the enrichment culture incubated for 24 h with ^{13}C-benzene showing all cells ($^{12}C^{14}N^-$), cells of phylotype BzS1 identified by specific probing ($^{19}F^-$) and the incorporation of ^{13}C into biomass by individual cells ($^{13}C/^{12}C$). Cells were marked in order to calculate the ^{13}C-enrichment of cells of phylotype BzS1 *vs*. cells belonging to other phylotypes. Scale bars, 2 µm.

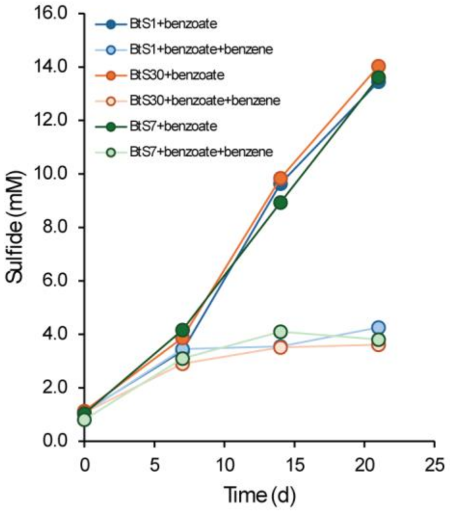

FIGURE S3 | Physiological experiments with benzoate-degrading strains isolated from the benzene-degrading, sulphate-reducing enrichment culture. Strains BtS1 and BtS30 (affiliating with *Desulfotignum*), and strain BtS7 (affiliating with *Desulfosarcina*) were grown with benzoate (5 mM, filled circles), or with a mixture of benzoate (1 mM) and benzene (0.5% v/v in HMN; open circles). In all cases, sulphide production was consistent with benzoate oxidation only.

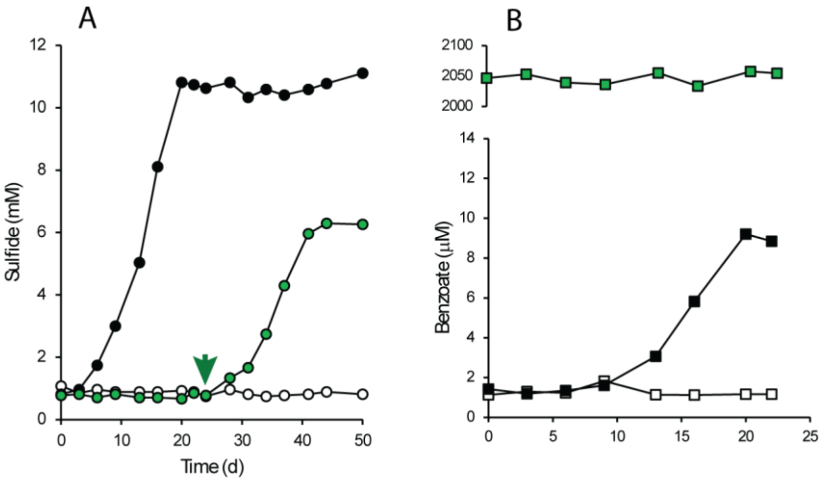

FIGURE S4 | Growth physiological experiments with sulphate-reducing strain BzS1. (A) Strain BzS1 grew anaerobically with benzene (filled black circles) but not with benzoate (filled green circles). Addition of benzene on incubation day 22 (arrowhead) led to immediate sulphide formation, proving cell viability. No sulphide formation was observed in the absence of added benzene (open circles). (B) During anaerobic growth with benzene, strain BzS1 excreted small amounts of benzoate (filled black squares). Benzoate added to the culture medium was not consumed (filled green squares). No benzoate was detected in cultures without growth-supporting benzene (open squares).

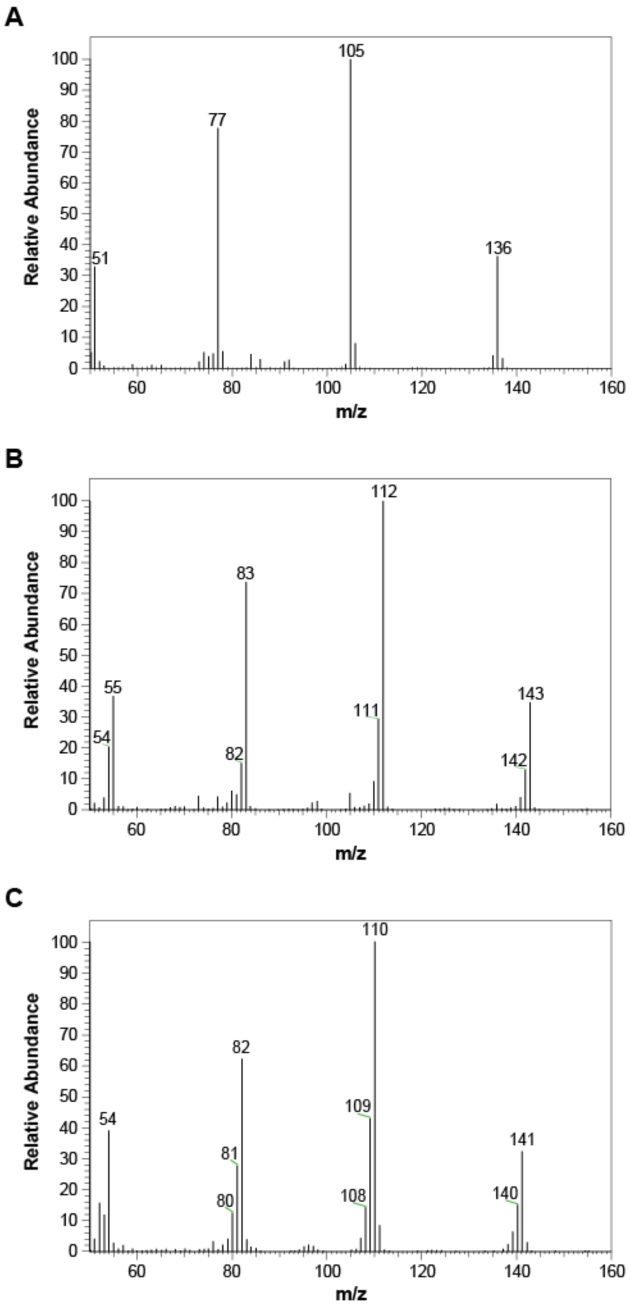

FIGURE S5 | Mass spectra of benzoates (as methyl esters) detected upon anaerobic growth of strain BzS1 with differently labelled substrates. (A) Unlabelled benzene. (B) ($^{13}C_6$)Benzene. (C) ($^{2}H_6$)Benzene. Key features of the observed labelling patterns are summarized in Figure 2.

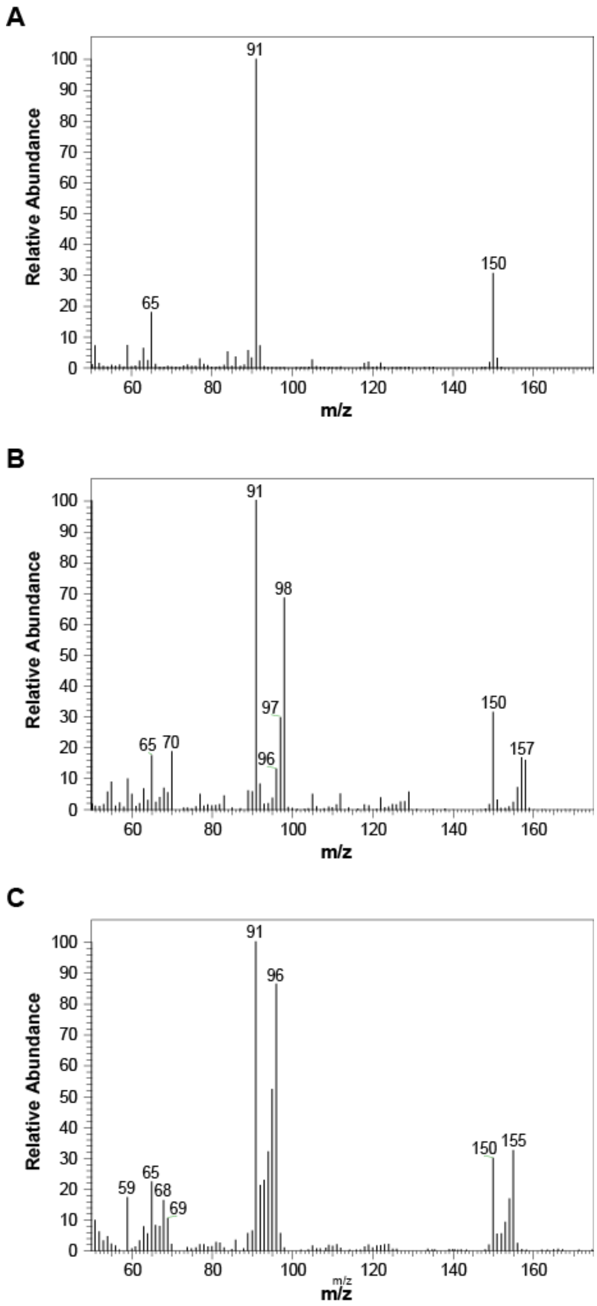

FIGURE S6 | Mass spectra of phenylacetates (as methyl esters) detected upon anaerobic growth of strain BzS1 with differently labelled substrates. (A) Unlabelled benzene. (B) ($^{13}C_6$)Benzene. (C) ($^{2}H_6$)Benzene. Key features of the observed labelling patterns are summarized in Figure 2. In contrast to detected benzoates (Figure S4) and 3-phenylpropanoates (Figure S6), the mass spectra in B and C reveal a significant proportion of unlabelled phenylacetate (M^+ at *m/z* 150, $[M\text{-}C_2H_3O_2]^+$ at *m/z* 91), whose occurrence cannot be explained at present.

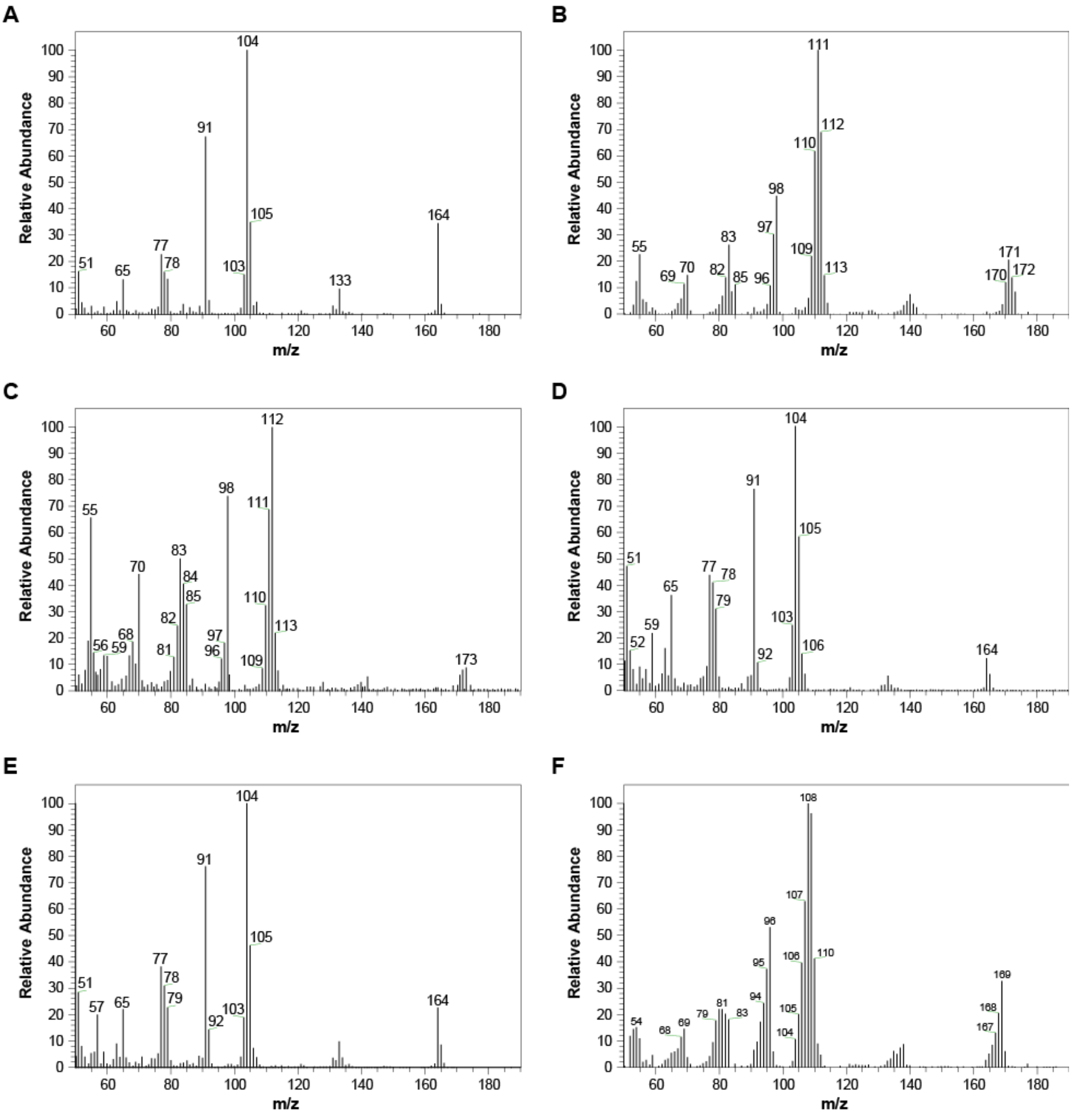

FIGURE S7 | Mass spectra of 3-phenylpropanoates (as methyl esters) detected upon anaerobic growth of strain BzS1 with differently labelled substrates. (A) Unlabelled benzene. (B) ($^{13}C_6$)Benzene. (C) ($^{13}C_6$)Benzene + unlabelled toluene. (D) Unlabelled benzene + 1-(^{13}C)acetate. (E) Unlabelled benzene + 2-(^{13}C)acetate. (F) ($^2H_6$)Benzene. Key features of the observed labelling patterns are summarized in Figure 2.

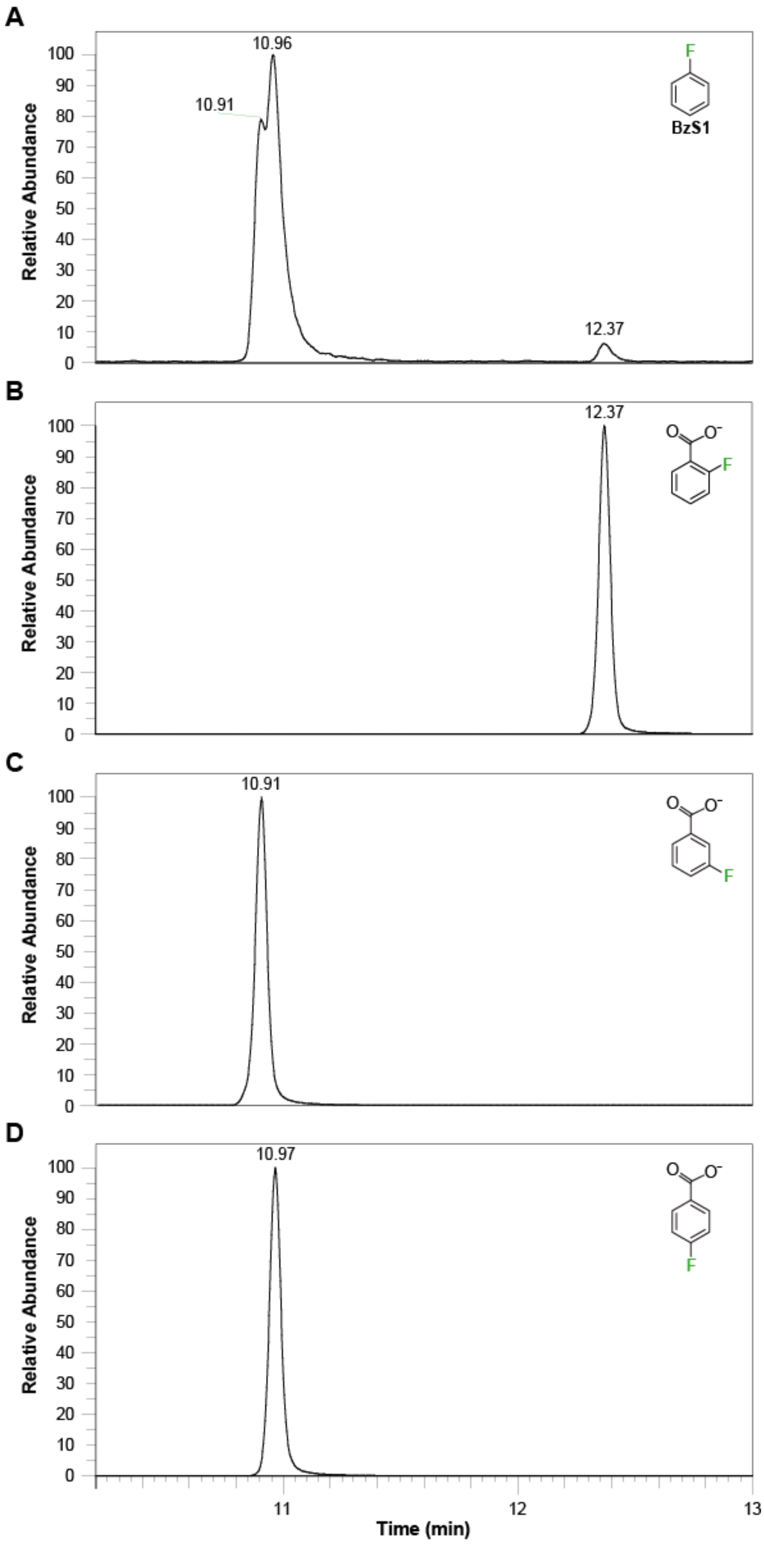

FIGURE S8 | Ion chromatograms (*m*/*z* = 154 + 123 + 95) revealing the elution order and relative abundance of the fluorobenzoates (as methyl esters) detected upon anaerobic growth of strain BzS1 with a mixture of benzene and fluorobenzene. (A) Culture extract. Reference standards: (B) 2-Fluorobenzoate, (C) 3-Fluorobenzoate, (D) 4-Fluorobenzoate.

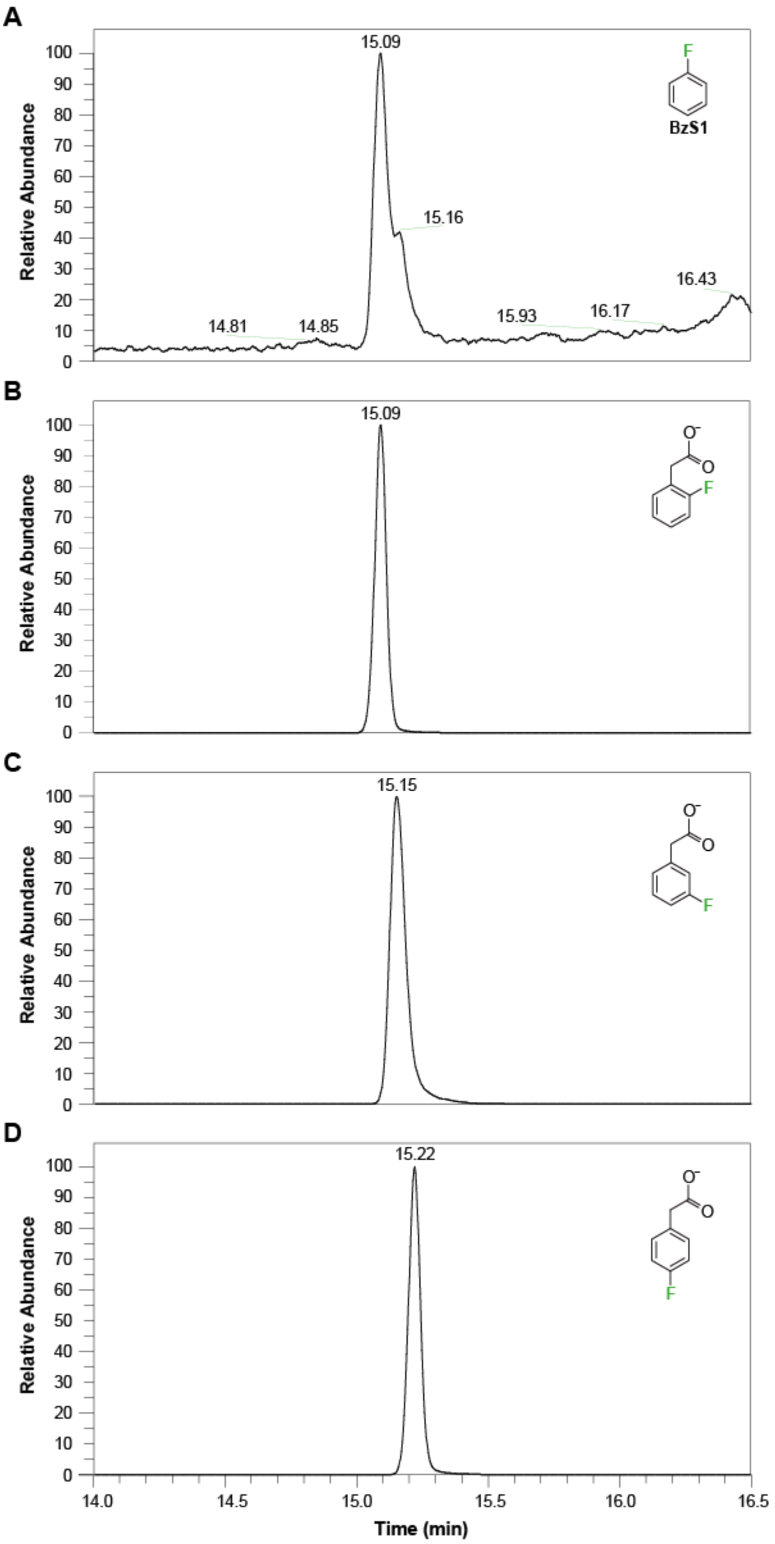

FIGURE S9 | Ion chromatograms (m/z = 168 + 109 + 83) revealing the elution order and relative abundance of the fluorophenylacetates (as methyl esters) detected upon anaerobic growth of strain BzS1 with a mixture of benzene and fluorobenzene. (A) Culture extract. Reference standards: (B) (2-Fluorophenyl)acetate, (C) (3-Fluorophenyl)acetate, (D) (4-Fluorophenyl)acetate.

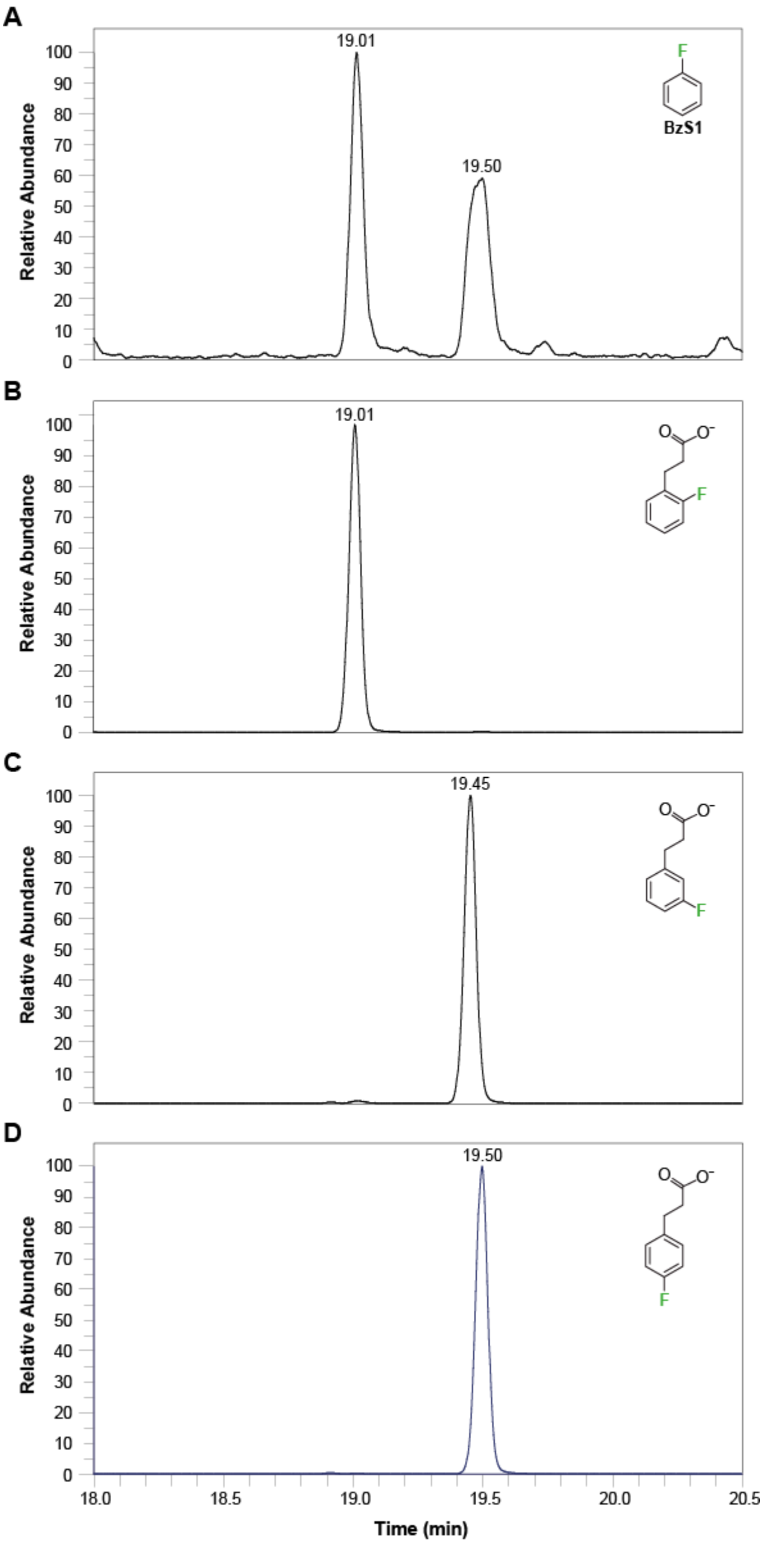

FIGURE S10 | Ion chromatograms (m/z = 182 + 123 + 109) revealing the elution order and relative abundance of the 3-(fluorophenyl)propanoates (as methyl esters) detected upon anaerobic growth of strain BzS1 with a mixture of benzene and fluorobenzene. (A) Culture extract. Reference standards: (B) 3-(2-Fluorophenyl)propanoate, (C) 3-(3-Fluorophenyl)propanoate, (D) 3-(4-Fluorophenyl)propanoate.

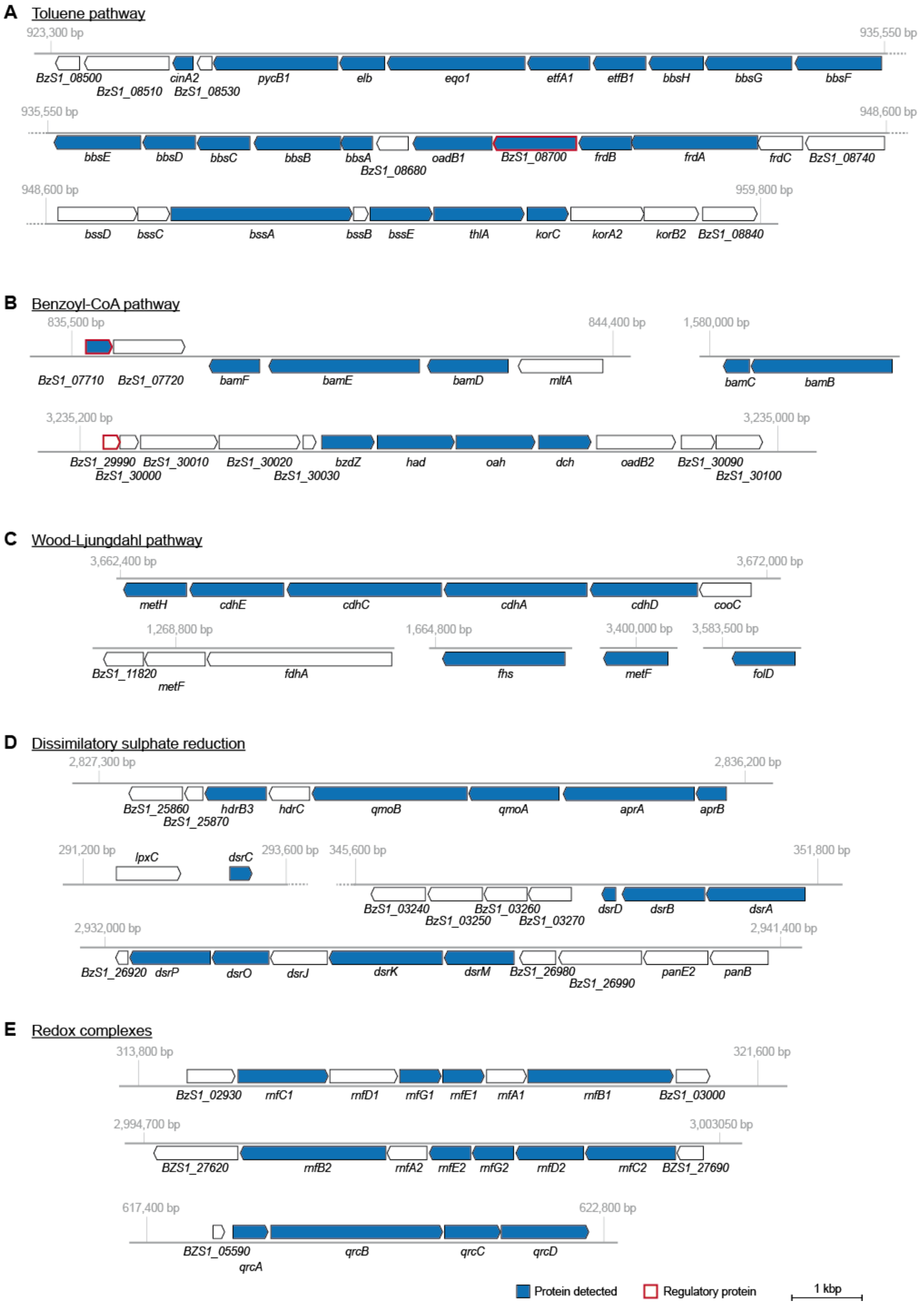

FIGURE S11 | Gene clusters and proteomic detection for selected metabolic processes in strain BzS1. (A) Anaerobic toluene degradation. (B) Central anaerobic benzoyl-CoA pathway. (C) Wood-Ljungdahl pathway. (D) Dissimilatory sulphate reduction. (E) Transmembrane redox complexes.

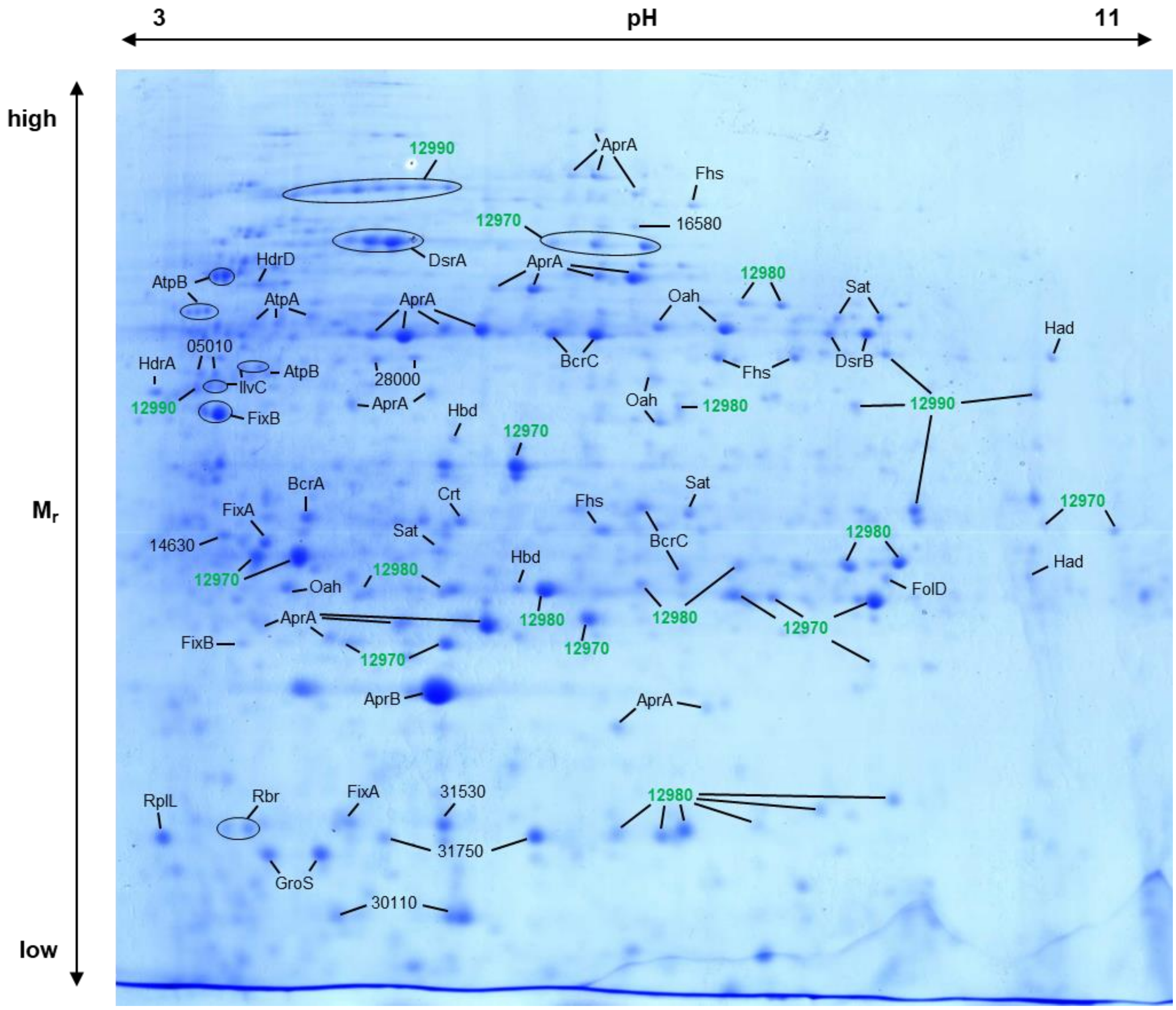

FIGURE S12 | Coomassie-stained 2DE-gel from the anaerobically benzene-degrading enrichment culture. Protein abbreviations resp. numbers are as follows (identification was based in Edman sequencing): AprAB, adenylylsulfate reductase; AtpAB, F0F1-type ATP synthase; BcrAC, benzoyl-CoA reductase; Crt, crotonase; DsrAB, dissimilatory sulphite reductase; Fhs, formate-tetrahydrofolate ligase; FixAB, electron transfer flavoprotein; FolD, methylenetetrahydrofolate dehydrogenase/cyclohydrolase; GroS, chaperonin; Had, 6-hydroxycyclohex-1-ene-1-carboxyl-CoA dehydrogenase; Hbd, 3-hydroxybutyryl-CoA dehydrogenase; HdrAD, heterodisulfide reductase; IlvC, ketol-acid reductoisomerase; Oah, 6-oxo-cyclohex-1-ene-carbonyl-CoA hydrolase; Sat, sulphate adenylyl-transferase; Rbr, rubrerythrin; RplL, ribosomal protein L7/L12; BzS1_05010, acyl-CoA dehydrogenase; **BzS1_12970, FAD-linked oxidase; BzS1_12980, similar to CoB-CoM heterodisulfide reductase; BzS1_12990, FAD-linked oxidase;** BzS1_14630, 4Fe-4S ferredoxin; BzS1_16580, 4Fe-4S ferredoxin; BzS1_28000, fructose-1,6-bisphosphatase; BzS1_30110, hypothetical protein; BzS1_31530, hypothetical protein; BzS1_31750, hypothetical protein.

SDS-PAGE

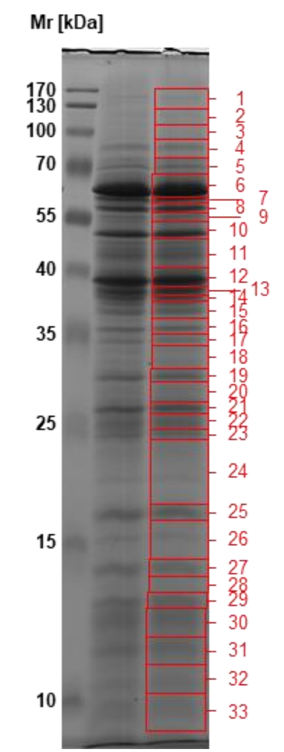

Slice no.	Top 5 protein identifications (locus tag; score)				
4	Similar to Hdr<br>(BzS1_25900; 10039)	CODH/Acetyl-CoA synthase<br>(BzS1_34070; 6340)	AprA, Adenylylsulfate reductase<br>(BzS1_25920; 4061)	FAD linked oxidase domain protein<br>(BzS1_12990; 1068)	Similar to CoB-CoM Hdr2<br>(BzS1_12980; 738)
5	DnaK, Chaperone<br>(BzS1_15980; 4205)	Peroxidase/catalase<br>(BzS1_15070; 3192)	AprA, Adenylylsulfate reductase<br>(BzS1_25920; 2850)	AcsA, Acetyl-CoA synthetase<br>(BzS1_09090; 2759)	FAD linked oxidase domain protein<br>(BzS1_12990; 1821)
6	AprA, Adenylylsulfate reductase<br>(BzS1_25920; 51395)	FAD linked oxidase domain protein<br>(BzS1_12990; 3987)	Aldehyde Fd oxidoreductase<br>(BzS1_14640; 3111)	CO dehydrogenase (acceptor)<br>(BzS1_34080; 2993)	DnaK, Chaperone<br>(BzS1_15980; 1808)
7	FAD linked oxidase domain protein<br>(BzS1_12990; 16727)	AprA, Adenylylsulfate reductase<br>(BzS1_25920; 12433)	Fhs, Formate-THF ligase<br>(BzS1_15450; 2991)	FAD-dep. oxidoreductase<br>(BzS1_33880; 2552)	FAD-dep. oxidoreductase<br>(BzS1_16610; 2279)
8	Fhs, Formate-THF ligase<br>(BzS1_15450; 10844)	AcsA, Acetyl-CoA synthetase<br>(BzS1_26210; 6220)	AprA, Adenylylsulfate reductase<br>(BzS1_25920; 3479)	FAD-dep. oxidoreductase<br>(BzS1_33880; 2207)	FAD linked oxidase domain protein<br>(BzS1_12990; 2042)
9	Uroporphyrin methyltransferase<br>(BzS1_18260; 2999)	Fhs, Formate-THF ligase<br>(BzS1_15450; 2738)	AprA, Adenylylsulfate reductase<br>(BzS1_25920; 1791)	4Fe-4S ferredoxin<br>(BzS1_16590; 1552)	AcsA, Acetyl-CoA synthetase<br>(BzS1_26210; 1312)
10	FAD linked oxidase domain protein<br>(BzS1_12970; 12728)	DsrA, Sulfite reductase<br>(BzS1_03300; 3231)	AprA, Adenylylsulfate reductase<br>(BzS1_25920; 3053)	Hypothetical protein<br>(BzS1_13420; 2539)	FAD linked oxidase domain protein<br>(BzS1_12990; 2322)
11	Cytochrome *c* precursor<br>(BzS1_24310; 4266)	Similar to CoB-CoM Hdr2<br>(BzS1_12980; 3833)	L-Carnitine dehydratase<br>(BzS1_26190; 3362)	Fumarate reductase<br>(BzS1_25910; 2606)	AprA, Adenylylsulfate reductase<br>(BzS1_25920; 2553)
12	Sat, Sulfate adenylyltransferase<br>(BzS1_08070; 12247)	Similar to CoB-CoM Hdr2<br>(BzS1_12980; 7529)	Acetyl-CoA acetyltransferase<br>(BzS1_15430; 3055)	CODH/Acetyl-CoA synthase<br>(BzS1_34060; 1811)	L-Carnitine dehydratase<br>(BzS1_26180; 1808)
13	Oah, Oxocyclohex..hydrolase<br>(BzS1_30060; 2652)	Acetyl-CoA acetyltransferase<br>(BzS1_20420; 2395)	Similar to CoB-CoM Hdr2<br>(BzS1_12980; 2386)	BcrC, Benzoyl-CoA reductase<br>(BzS1_02010; 1388)	Sat, Sulfate adenylyltransferase<br>(BzS1_08070; 1255)
14	Acyl-CoA dehydogenase<br>(BzS1_05010; 7762)	DsrB, Sulfite reductase<br>(BzS1_03290; 5156)	AprA, Adenylylsulfate reductase<br>(BzS1_25920; 5087)	Had, 6-Hydroxycyclo....dehydrogen<br>(BzS1_30050; 3221)	Oah, 6-Oxo-cyclohex....hydrolase<br>(BzS1_30060; 2986)
15	IlvC, Ketol-acid reductoisomerase<br>(BzS1_02780; 4361)	Had, 6-Hydroxycyclo....DH<br>(BzS1_30050; 3898)	AprA, Adenylylsulfate reductase<br>(BzS1_25920; 3815)	Fbp, Fructose-1,6-bisphosphatase<br>(BzS1_28000; 3726)	Aspartate-semialdehyde DH<br>(BzS1_05460; 2539)
16	Hbd, 3-Hydroxybutyryl-CoA DH<br>(BzS1_13040; 11370)	AprA, Adenylylsulfate reductase<br>(BzS1_25920; 2646)	Chaperone GroEL<br>(BzS1_02880; 2322)	Phosphoserine aminotransferase<br>(BzS1_10390; 1838)	Oah, 6-Oxo-cyclohex….hydrolase<br>(BzS1_30060; 1471)
17	FixB, Electron transfer flavoprotein<br>(BzS1_21400; 4275)	Gap, Glyceraldehyde-3-P-DH<br>(BzS1_14490; 2884)	AprA, Adenylylsulfate reductase<br>(BzS1_25920; 2302)	Hbd, 3-Hydroxybutyryl-CoA DH<br>(BzS1_13040; 1586)	Oah, 6-Oxo-cyclohex….hydrolase<br>(BzS1_30060; 1096)
19	Dihydropteroate synthase<br>(BzS1_34050; 4655)	FolD, Methylene-THF-DH<br>(BzS1_33310; 985)	AprA, Adenylylsulfate reductase<br>(BzS1_25920; 232)	Acetyl-CoA acetyltransferase<br>(BzS1_15430; 143)	L-seryl-tRNA(Sec) selenium transf.<br>(BzS1_08350; 104)
21	Conserved hypothetical protein<br>(BZS1_27730; 14635)	Crt, Crotonase<br>(BzS1_13050; 1910)	Alpha/beta hydrolase fold<br>(BzS1_23250; 1883)	Chaperone GroEL<br>(BzS1_02880; 1598)	MvhD, Methylviologen-red. hydrog<br>(BzS1_07730; 1457)
22	Crt, Crotonase<br>(BZS1_13050; 3913)	BzdZ, Putative dehydrogenase<br>(BzS1_30040; 3244)	Conserved hypothetical protein<br>(BzS1_27730; 3216)	AprA, Adenylylsulfate reductase<br>(BzS1_25920; 3118)	CaiD, Carnithine racemase<br>(BzS1_21900; 3085)
23	Adk, Adenylate kinase<br>(BZS1_33730; 5272)	BzdZ, Putative dehydrogenase<br>(BzS1_30040; 2300)	TpiA, Triosephosphate isomerase<br>(BzS1_14500; 2284)	Crt, Crotonase<br>(BzS1_13050; 1716)	AprA, Adenylylsulfate reductase<br>(BzS1_25920; 1000)
25	AprB, Adenylylsulfate reductase<br>(BzS1_25930; 2039)	Periplasmic binding protein<br>(BzS1_18270;153)	Rbr, Rubrerythrin<br>(BzS1_32110; 100)	HslV, ATP-dep. protease<br>(BzS1_30680; 76)	Hypothetical protein<br>(BzS1_31520; 74)
27	Hypothetical protein<br>(BzS1_31520; 2272)	MoaB, Molybdenum....biosynthesis<br>(BzS1_13690; 192)	Hypothetical protein<br>(BzS1_30110; 182)	AprA, Adenylylsulfate reductase<br>(BzS1_25920; 168)	AprB, Adenylylsulfate reductase<br>(BzS1_25930; 114)

FIGURE S13 | Coomassie-stained 1DE-gel from soluble fraction of the anaerobically benzene-degrading enrichment culture with top five protein identifications per analysed gel slice.

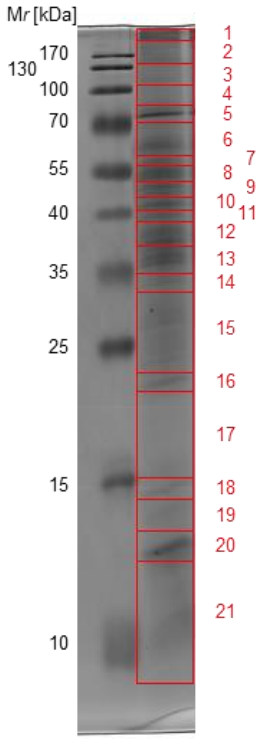

Slice no.	Top 3 protein identifications (locus tag; score)			FAD linked oxidase domain protein (prot_hit_no.; score) BzS1_12970	BzS1_12990	Hdr-like (see left) BzS1_12980
1	HppA, (H(+)-PPase) (BzS1_05800; 846)	Possible toxin (BzS1_23370; 411)	hypothetical protein (BzS1_28970; 315)	n.d.	(19 of 50; 37)	n.d.
2	HppA, (H(+)-PPase) (BzS1_05800; 2561)	4Fe-4S ferredoxin (BzS1_16530; 1242)	4Fe-4S ferredoxin (BzS1_03120; 1171)	(18 of 100; 196)	(29 of 100; 108)	n.d.
3	HdrA, Fe/S subunit A (BzS1_03160; 1863)	HppA, (H(+)-PPase) (BzS1_05800; 1313)	HdrA, fragement (BzS1_07740; 682)	n.d.	(22 of 100; 155)	(41 of 100; 53)
4	Outer membrane protein (BzS1_01750; 815)	HdrA, Fe/S subunit A (BzS1_03160; 812)	CODH/ACS, beta subunit (BzS1_34070; 754)	n.d.	(26 of 100; 241)	n.d.
5	TonB-dependent receptor (BzS1_18250; 5915)	Mo oxidoreductase (BzS1_05610; 4224)	RnfB (BzS1_27630; 3354)	(33 of 100; 82)	(19 of 100; 165)	(46 of 100; 46)
6	AprA, (BzS1_25920; 4566)	FAD linked oxidase (BzS1_12990; 3342)	Aldehyde Fd oxidoreductase (BzS1_14640; 2406)	(16 of 100; 353)	(2 of 100; 3342)	(28 of 100; 150)
7	Fhs (BzS1_15450; 6089)	AcsA, Acetyl-CoA synth. (BzS1_26210; 2436)	Putative biosynthesis protein (BzS1_33050; 1812)	(15 of 100; 562)	(5 of 100; 1785)	(33 of 100; 159)
8	Hypothetical protein (BzS1_13410; 2937)	Peptidyl isomerase (BzS1_33670; 2674)	Fhs (BzS1_15450; 2663)	(8 of 100; 1707)	(9 of 100; 1495)	(37 of 100; 130)
9	FAD linked oxidase (BzS1_12970; 5097)	RnfC (BzS1_27680; 3573)	Outer membrane efflux (BzS1_08150; 2065)	(1 of 100; 5097)	(17 of 100; 519)	(22 of 100; 359)
10	MotA/TolQ/ExbB (BzS1_18200; 4436)	hypothetical protein (BzS1_04550; 2670)	AtpB (BzS1_31240; 2517)	(20 of 100; 504)	(22 of 100; 481)	(14 of 100; 697)
11	hypothetical protein (BzS1_31050; 3132)	Hdr-like (BzS1_12980; 2343)	HlyD (secretion) (BzS1_08160; 2012)	(7 of 100; 1081)	(18 of 100; 388)	(2 of 100; 2343)
12	Hdr-like (BzS1_12980; 4046)	OmpA (BzS1_00220; 2721)	hypothetical protein (BzS1_27800; 2219)	(7 of 100; 1459)	(34 of 100; 181)	(1 of 100; 4046)
13	HlfK (BzS1_12840; 2454)	Had (BzS1_30050; 2065)	Hdr-like (BzS1_12980; 1791)	(18 of 100; 372)	(29 of 100; 185)	(3 of 100; 1791)
14	AtpG (BzS1_31250; 4412)	Polysaccharide export (BzS1_33060; 2430)	Hbd (BzS1_13040; 1380)	(15 of 100; 264)	(22 of 100; 119)	(7 of 100; 884)
15	HlfC (BzS1_12850; 1652)	RplB (BzS1_26640; 1564)	FliC (BzS1_04840; 973)	(35 of 100; 103)	(39 of 100; 80)	(6 of 100; 644)
16	Protein H secretion (BzS1_16870; 1808)	hypothetical protein (BzS1_16400; 1411)	RpsD (BzS1_26430; 1014)	(25 of 100; 203)	(38 of 100; 76)	(12 of 100; 430)
17	hypothetical protein (BzS1_28970; 1 229)	RnfG (BzS1_27660; 770)	hypothetical protein (BzS1_23790; 614)	(73 of 100; 32)	(43 of 100; 83)	(17 of 100; 275)
18	Outer membrane protein (BzS1_01930; 1110)	hypothetical protein (BzS1_28970; 1054)	biosynthesis protein (BzS1_28440; 967)	(48 of 100; 34)	(30 of 100; 61)	(26 of 100; 98)
19	biosynthesis protein (BzS1_28440; 2144)	AtpF (BzS1_17290; 1120)	RpsE (BzS1_26500; 1087)	n.d.	n.d.	(29 of 100; 111)
20	hypothetical protein (BzS1_31750; 3277)	hypothetical protein (BzS1_28970; 1779)	AtpG (BzS1_17300; 1336)	(24 of 100; 204)	(45 of 100; 46)	(38 of 100; 76)
21	hypothetical protein (BzS1_28970; 1922)	RplV (BzS1_26620; 904)	hypothetical protein (BzS1_31750; 842)	n.d.	n.d.	(69 of 100; 36)

FIGURE S14 | Coomassie-stained SDS-gel from the membrane protein-enriched fraction of the anaerobically benzene-degrading enrichment culture with top three protein identifications per analysed gel slice, highlighting those of the FAD-linked oxidase like proteins and the HDr-like protein (green). Note, that according to identification scores their relative abundances in the membrane fraction might be according to the order Hdr-like protein (BzS1_12980) > FAD-linked oxidase (BzS1_12970) > FAD-linked oxidase (BzS1_12990).

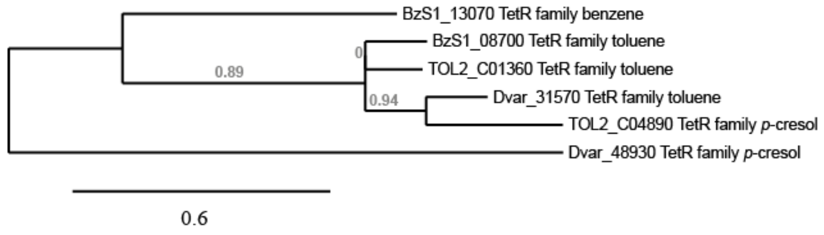

FIGURE S15 | Phylogenetic clustering of TetR family regulator (BzS1_13070) from BzS1.

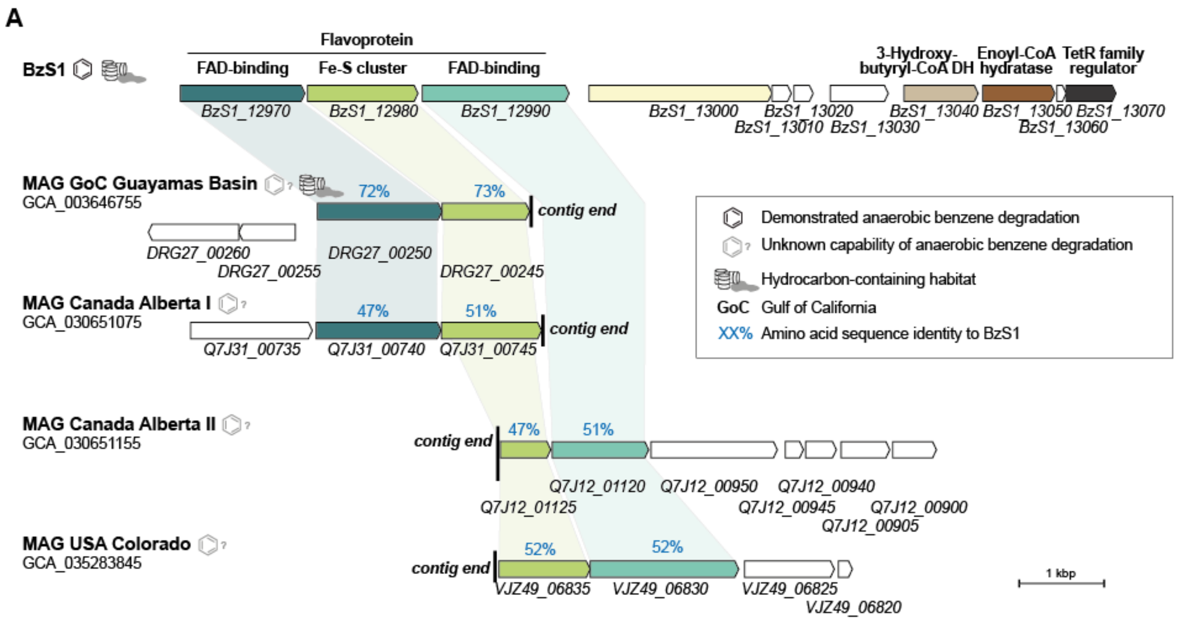

B

Organism/MAG	Accession	Size (Mbp)	Location	Isolation source	Latitude and longitude
BzS1	Acc	3.7	France, Etang de Berre	Contaminated marine sediment	43.485 N 5.170 E
MAG GoC Pescadero Basin	GCA_021163815	1.6	Mexico, Gulf of California, Pescadero Basin	Hydrothermal vent sediment	23.9570 N 108.8622 W
Ca. Pelotomaculum BPL	IMG 62780	3.0	Poland, Gliwice	Coal gasification site	50.3004 N 18.6572 E
Ca. Benzenivorax ORM2a	CP113000	3.3	USA, Oklahoma	Oil contaminated soil	35 N 97 W
MAG China South China Sea	GCA_020351915	5.1	China, South China Sea	Marine sediment	22.1315 N 113.8055 E
MAG Israel Haifa	GCA_034190765	4.4	Israel, Haifa	Tar patties	32.826 N 34.956 E
Chloroflexota bacterium	GCA_042575365	1.0	USA, Maine	Marine sediment	43.994827 N 69.6486 W
Dehalococcides bacterium	GCA_055098835	1.0	China, Shenzhen	Mangrove wetland sediment	22.5288 N 114.0030 E
Deltaproteobacterium NaphS2	GCA_000179315	6.6	Germany, Horumersiel	Marine sediment	53.6857 N 8.0236 E
Ds. variabilis 3be13	GCA_048565275	9.6	France, Montpeliier	Marine sediment	43.5194 N 3.8996 E
MAG GoC Guayamas Basin	GCA_003646755	1.2	Mexico, Gulf of California, Guaymas Basin	Marine sediment	29.5241 N 113.5700 W
MAG Canada Alberta I	GCA_030651075	3.7	Canada, Alberta	Groundwater	55.45 N 111.33 W
MAG Canada Alberta II	GCA_030651155	1.7	Canada, Alberta	Groundwater	55.45 N 111.33 W
MAG USA Colorado	GCA_035283845	3.2	USA, Colorado	Soil	39.5347 N 107.7826 W

FIGURE S16 | Additional comparative analyses of the abundant, putatively heterotrimeric flavoprotein from BzS1. Gene clusters (A) of further putative flavoprotein subunits and geographical origin (B) of compared strains, cultures and MAGs (including those shown in Figure 5).

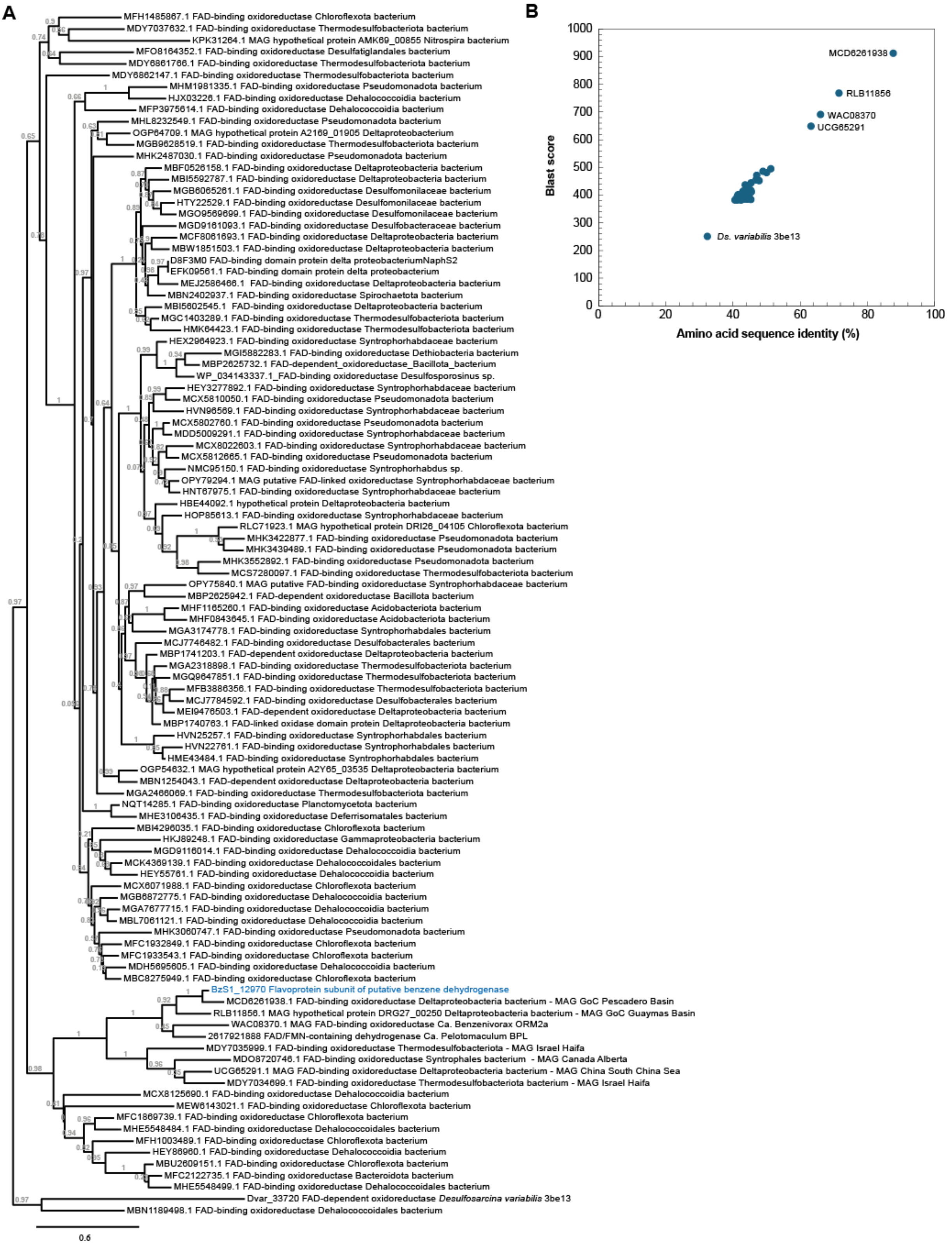

FIGURE S17 | Phylogenetic affiliations of putative flavoprotein subunit BzS1_12970. Accession numbers of protein sequences are indicated. For underlying top 100 BLAST-results refer to Table S4.

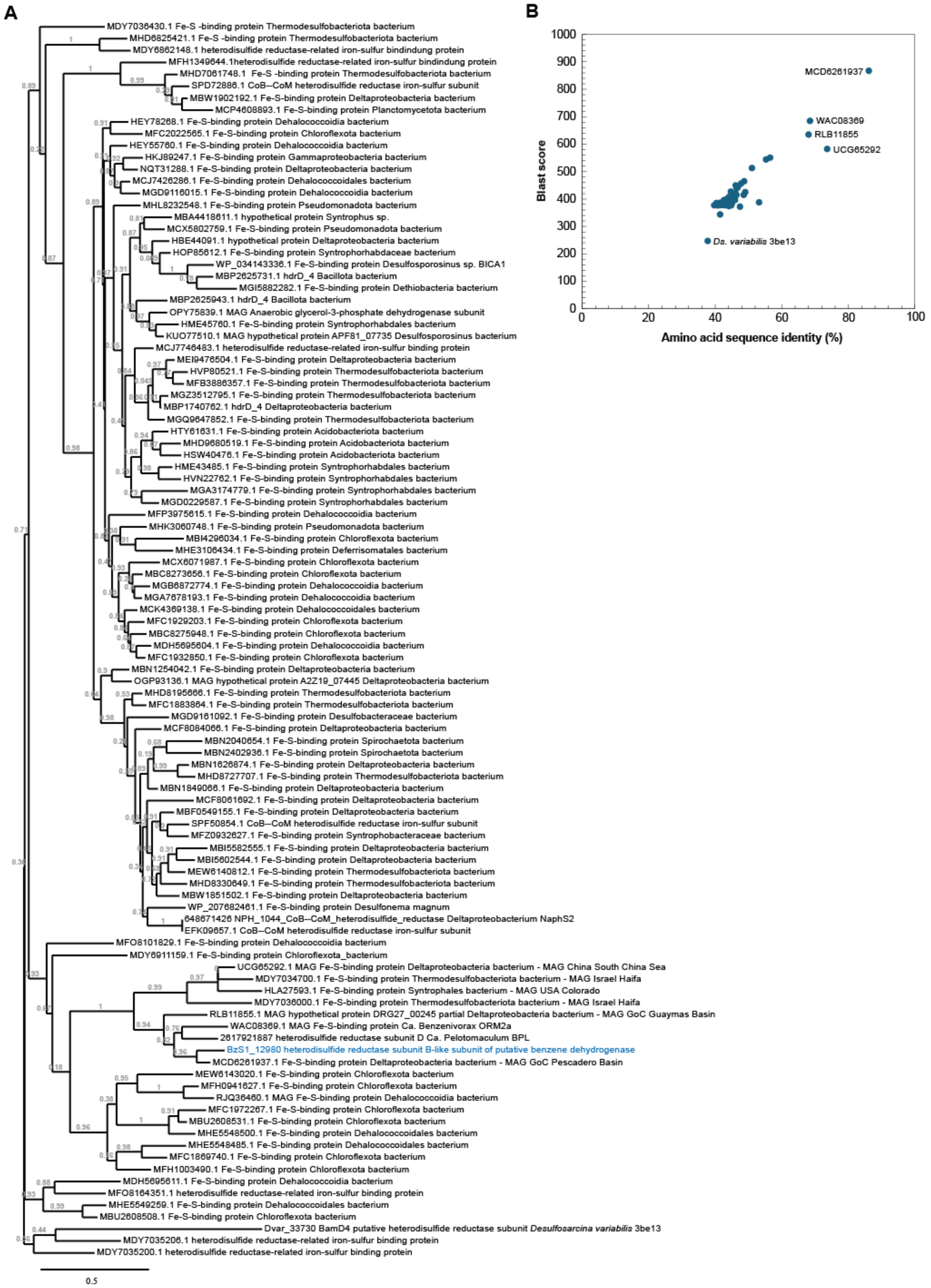

FIGURE S18 | Phylogenetic affiliations of putative flavoprotein subunit BzS1_129780. Accession numbers of protein sequences are indicated. For underlying top 100 BLAST-results refer to Table S4.

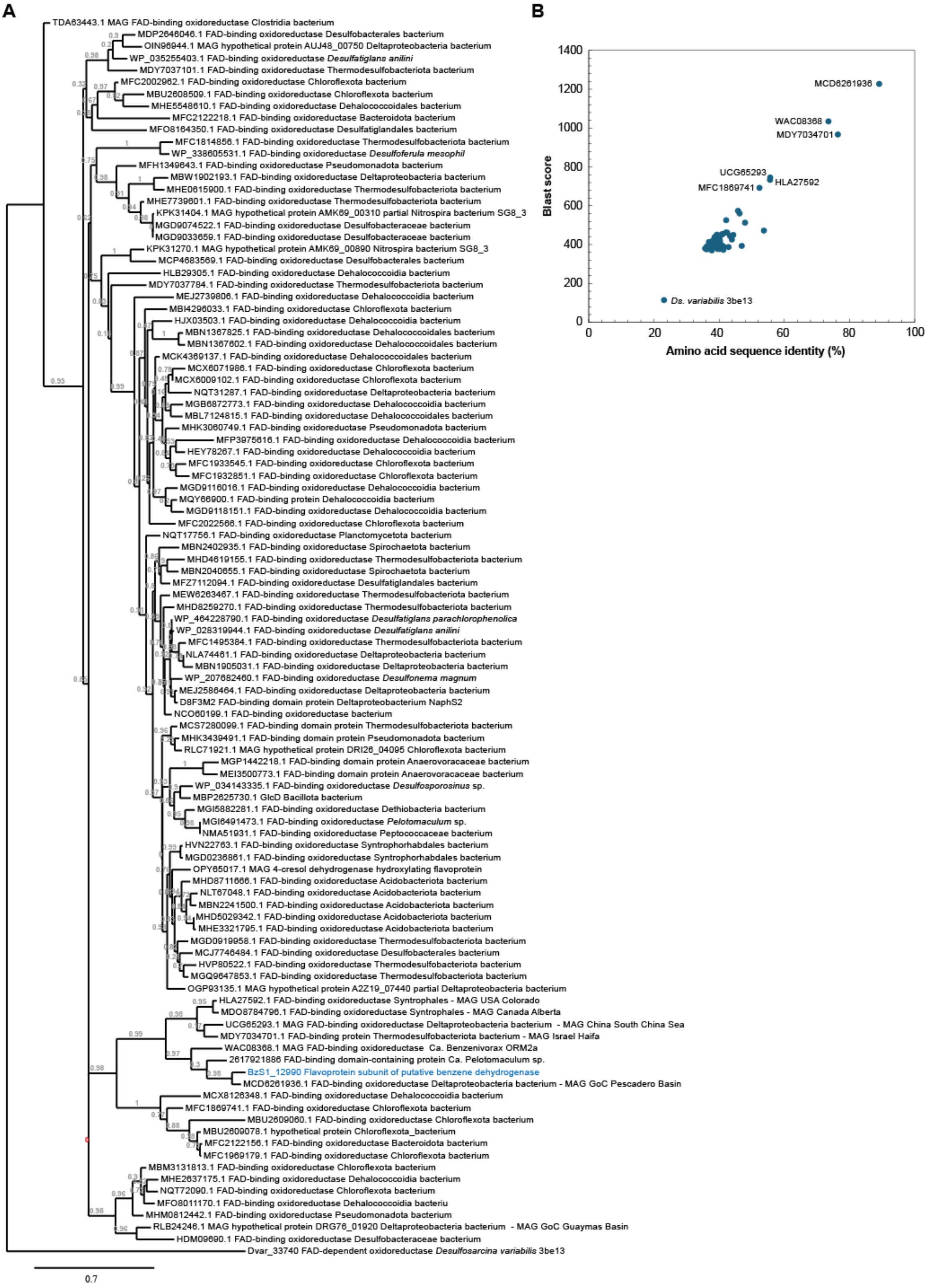

FIGURE S19 | Phylogenetic affiliations of putative flavoprotein subunit BzS1_12990. Accession numbers of protein sequences are indicated. For underlying top 100 BLAST-results refer to Table S4.

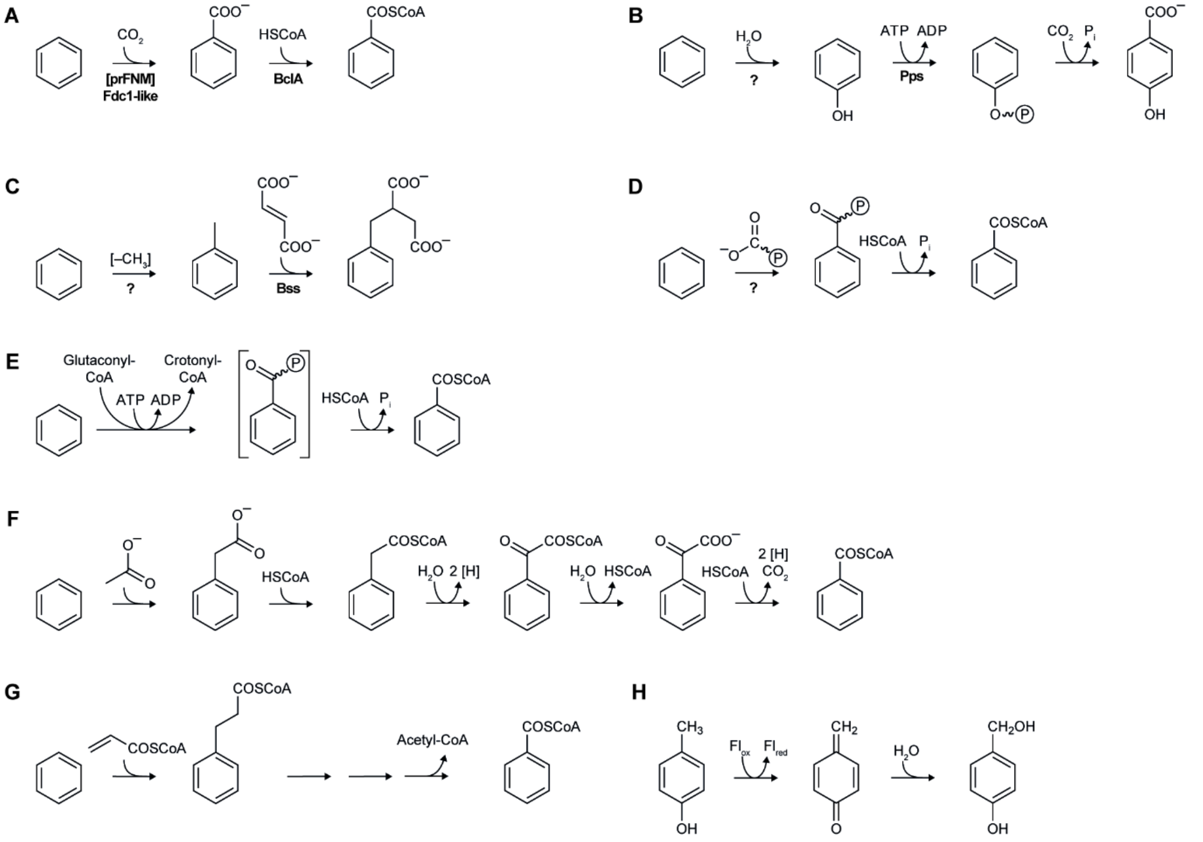

FIGURE S20 | Previously hypothesised and other conceivable reactions for the initial step(s) of anaerobic benzene degradation and further potentially relevant reactions. (A) 1,3-Cycloaddition of CO_2 via a preFMN-dependent carboxylation. (B) Involvement of a phenylphosphate carboxylase-like carboxylation reaction. (C) Methylation of benzene to toluene followed by formation of benzylsuccinate. (D) Addition of carboxyphosphate forming benzoyl-phosphate. (E) Transcarboxylation of benzene with glutaconyl-CoA. (F) Addition of acetate followed by phenylacetate degradation via α-oxidation. (G) Addition of benzene to acrylyl-CoA forming phenylpropanoyl-CoA. (H) *p*-Cresol oxidation to *p*-hydroxybenzyl alcohol by the flavocytochrome *c* enzyme *p*-cresol methylhydroxylase.

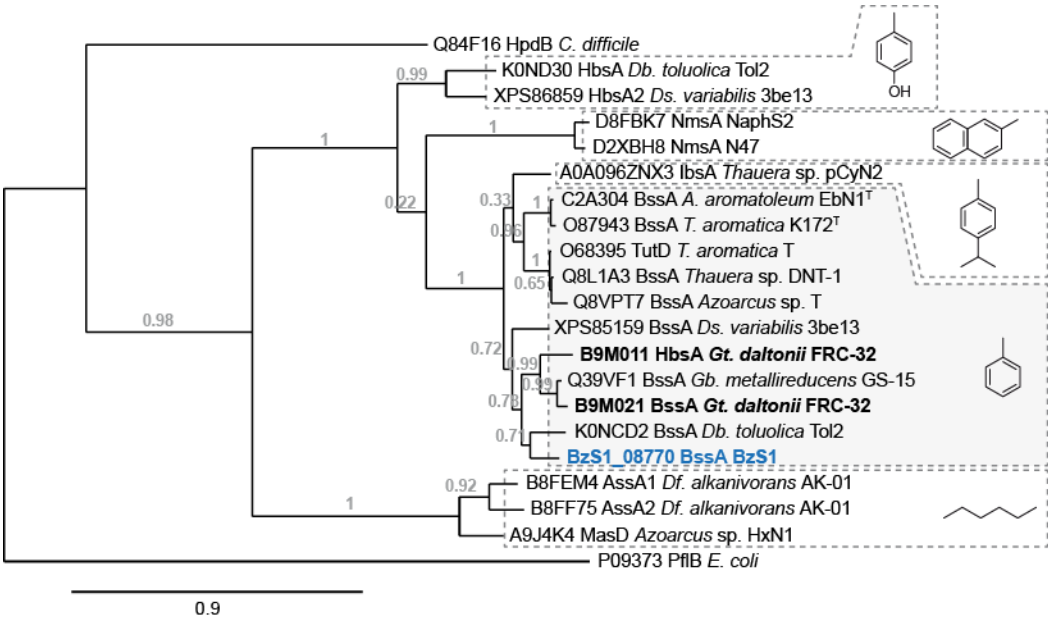

FIGURE S21 | Phylogenetic relations of catalytic subunits from aryl-/alkylsuccinate synthases. BssA from strain BzS1 is highlighted in bold blue, while BssA and HbsA from *Geotalea daltonii* are highlighted in bold black. All three proteins clearly fall into the cluster of toluene-activating enzymes. Furthermore, the *hbs* operon co-localizes with the *bss* and *bbs* operons in the genome of *G. daltonii*. By contrast, the catalytic subunit of *p*-cresol activating hydroxybenzylsuccinate synthases (HbsA) from the sulphate-reducing bacteria *Desulfobacula toluolica* Tol2 and *Desulfosarcina variabilis* cluster far apart (top). In addition, their *hbs* operons co-localize with the respective *bhs* operons (β-oxidation of (4-hydroxybenzyl)succinate to 4-hydroxylbenzoyl-CoA) (e.g., Wöhlbrand et al. 2013). The genome of *G. daltonii* does apparently not possess a *bhs* operon. Thus, these phylogenetic and genomic data suggest that Hbs of *G. daltonii* is a paralogous, toluene-activating enzyme.

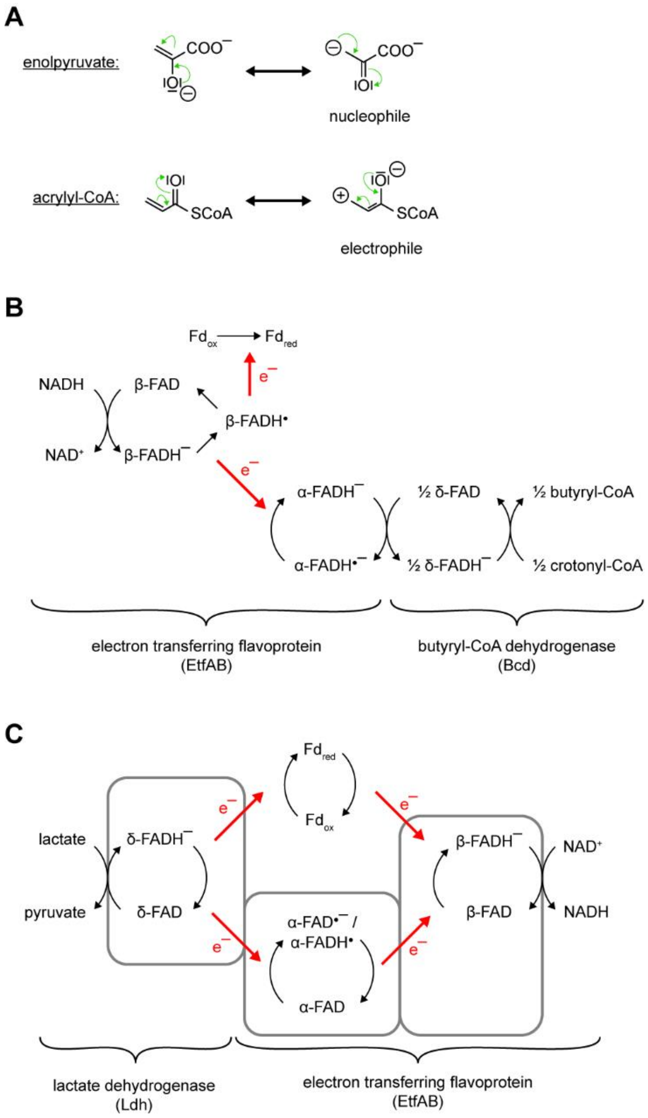

FIGURE S22 | Additional information on possible role of presumptively heterotrimeric flavoprotein BZS1_12970/80/90 in anaerobic benzene degradation by strain BzS1. (A) Resonance structures of enolpyruvate and acrylyl-CoA. (B) Flavin-based electron bifurcation (FBEB) as exemplified for the bifurcating electron transferring flavoprotein/butyryl-CoA dehydrogenase ((EtfAB-Bcd)$_4$) complex; modified from Demmer et al. (2017). (C) Flavin-based electron bifurcation/confurcation (FBEB/FBEC) as exemplified for the lactate dehydrogenase – electron transferring flavoprotein (Ldh-EtfAB) complex; modified from Kayastha et al. (2022).

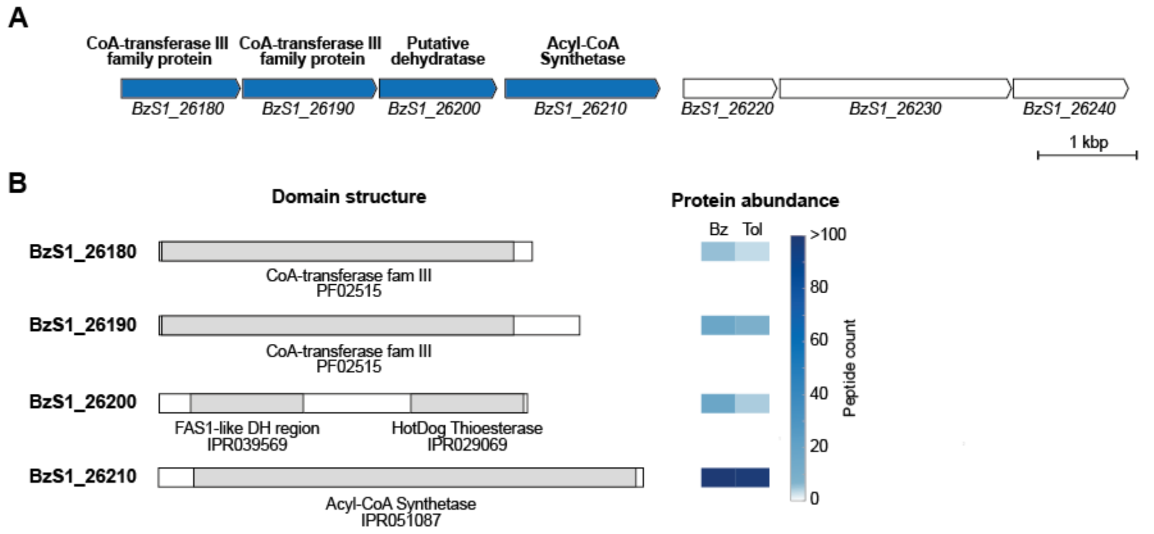

C

	Best SWISSPROT BLAST hit						
**BzS1 protein**	**Accession**	**Protein**	**Organism**	**Score**	**E-value**	**Identity (%)**	**Pos. (%)**
BzS1_16180	O87838	Formyl-CoA:oxalate CoA-transferase	*Streptomyces coelicolor*	189	10^{-54}	31	49
BzS1_16190	A9WGE3	Succinyl-CoA–D-citramalate CoA-transferase	*Chloroflexus aurantiacus*	208	10^{-61}	30	52
BzS1_16200	Q82DR8	UPF0336 protein	*Streptomyces avermitilis*	50	3×10^{-6}	33	45
BzS1_16210	P39062	Acetyl-coenzyme A synthetase	*Bacillus subtilis*	265	2×10^{-80}	30	51

FIGURE S23 | Possible candidate for α-elimination of water from 2-hydroxy-3-phenylpropanoyl-CoA encoded in the genome of strain BzS1. (A) Gene cluster. (B) Domain structure and detected protein abundances. (C) Functional annotation.

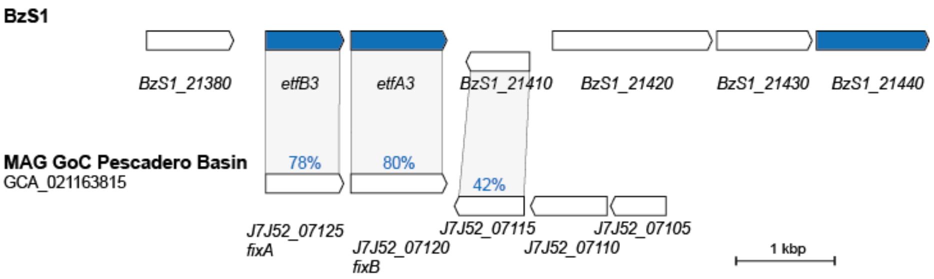

FIGURE S24 | Genomic context of electron transfer protein (EtfA3B3) present at high abundance in benzene-utilizing cells of strain BzS1 and comparison of coding genes with orthologs in the MAG from hydrocarbon-rich, hydrothermal marine sediment (MAG GoC Pescadero Basin).
